# Breakthrough Infections in SARS-CoV-2-Vaccinated Multiple Myeloma Patients Improve Cross-Protection against Omicron Variants

**DOI:** 10.3390/vaccines12050518

**Published:** 2024-05-09

**Authors:** Angelika Wagner, Erika Garner-Spitzer, Claudia Auer, Pia Gattinger, Ines Zwazl, René Platzer, Maria Orola-Taus, Peter Pichler, Fabian Amman, Andreas Bergthaler, Johannes B. Huppa, Hannes Stockinger, Christoph C. Zielinski, Rudolf Valenta, Michael Kundi, Ursula Wiedermann

**Affiliations:** 1 Institute of Specific Prophylaxis and Tropical Medicine, Center of Pathophysiology, Infectiology and Immunology, Medical University Vienna, 1090 Vienna, Austria; angelika.wagner@meduniwien.ac.at (A.W.); erika.garner-spitzer@meduniwien.ac.at (E.G.-S.); claudia.auer@meduniwien.ac.at (C.A.); ines.zwazl@meduniwien.ac.at (I.Z.); maria.orola-taus@meduniwien.ac.at (M.O.-T.); peter.pichler@meduniwien.ac.at (P.P.); 2 Center for Pathophysiology, Infectiology and Immunology, Department of Pathophysiology and Allergy Research, Medical University of Vienna, 1090 Vienna, Austria; pia.gattinger@meduniwien.ac.at (P.G.); rudolf.valenta@meduniwien.ac.at (R.V.); 3 Center of Pathophysiology, Infectiology and Immunology, Institute for Hygiene and Applied Immunology, Medical University Vienna, 1090 Vienna, Austria; rene.platzer@meduniwien.ac.at (R.P.); fabian.amman@meduniwien.ac.at (F.A.); andreas.bergthaler@meduniwien.ac.at (A.B.); johannes.huppa@meduniwien.ac.at (J.B.H.); hannes.stockinger@meduniwien.ac.at (H.S.); 4 Research Center for Molecular Medicine of the Austrian Academy of Sciences, CeMM, 1090 Vienna, Austria; 5 Wiener Privatklinik, and Central European Cooperative Oncology Group (CECOG), Central European Cancer Center, 1090 Vienna, Austria; christoph.zielinski@cancer-center.cc; 6 Karl Landsteiner University of Health Sciences, 3500 Krems, Austria; 7 Center for Public Health, Medical University Vienna, 1090 Vienna, Austria; michael.kundi@meduniwien.ac.at

**Keywords:** SARS-CoV-2 vaccination, multiple myeloma, immune response, immunosuppression, breakthrough infection, immune cell depletion, B memory cells

## Abstract

Patients with multiple myeloma (MM) are a heterogenous, immunocompromised group with increased risk for COVID-19 morbidity and mortality but impaired responses to primary mRNA SARS-CoV-2 vaccination. The effects of booster vaccinations and breakthrough infections (BTIs) on antibody (Ab) levels and cross-protection to variants of concern (VOCs) are, however, not sufficiently evaluated. Therefore, we analysed humoral and cellular vaccine responses in MM patients stratified according to disease stage/treatment into group (1) monoclonal gammopathy of undetermined significance, (2) after stem cell transplant (SCT) without immunotherapy (IT), (3) after SCT with IT, and (4) progressed MM, and in healthy subjects (prospective cohort study). In contrast to SARS-CoV-2 hu-1-specific Ab levels, Omicron-specific Abs and their cross-neutralisation capacity remained low even after three booster doses in a majority of MM patients. In particular, progressed MM patients receiving anti-CD38 mAb and those after SCT with IT were Ab low responders and showed delayed formation of spike-specific B memory cells. However, MM patients with hybrid immunity (i.e., vaccination and breakthrough infection) had improved cross-neutralisation capacity against VOCs, yet in the absence of severe COVID-19 disease. Our results indicate that MM patients require frequent variant-adapted booster vaccinations and/or changes to other vaccine formulations/platforms, which might have similar immunological effects as BTIs.

## 1. Introduction

Multiple myeloma (MM) is a plasma cell malignancy that develops from a pre-malignant stage, i.e., monoclonal gammopathy of unknown significance (MGUS), and can progress to smouldering (asymptomatic) MM and eventually to symptomatic MM. According to MM classification and disease stage, the treatment options include use of immunomodulatory drugs (such as lenalidomide and pomalidomide) and/or monoclonal antibodies (mAbs) directed at surface markers of malignant cells, as well as autologous stem cell transplantation (SCT) [1].

Immunosuppression in patients with haematological malignancies results from immune dysfunction due to disease as well as respective treatment. Therefore, MM patients in particular are at increased risk for COVID-19-related morbidity and mortality [2,3]. In our previously conducted cohort study with different immunocompromised subjects (i.e., MM and inflammatory bowel disease [IBD] patients), we analysed the immune responses to primary mRNA SARS-CoV-2 vaccination and identified both complete antibody (Ab) non-responders and patients with early Ab waning [4], as also shown by others [5,6,7]. Furthermore, in our cohort of IBD vaccinees, we demonstrated that the type of treatment strongly influenced the development of immunological memory [8]. As MM patients with plasma cell-targeting therapies (anti-CD38 and anti-B-Cell-Maturation-Antigen [BCMA] mAbs) showed very weak primary vaccine responses [5], the investigation of Ab kinetics and specific B memory cell development during booster vaccinations over a follow-up period of two years is of high interest.

Also due to the replacement of ancestral hu-1 virus by antigenically highly diverse Omicron variants in 2021 and the use of adapted of SARS-CoV-2 vaccines as of fall 2022, it is to date unclear how well this group is protected from infection and severe COVID-19 disease after vaccination. Therefore, we investigated (i) the kinetics of long-term SARS-CoV-2 Ab responses up to one month after the fifth dose in MM patients compared to controls, (ii) the cross-neutralisation capacity of monovalent hu-1 and bivalent hu-1/Omicron vaccine-induced Abs against Omicron BA.4/5 and XBB.1.5 strains, and (iii) the kinetics of spike (S)-specific B memory cell development. Furthermore, we wanted to know how MM disease stage and treatment modalities as well as occurring breakthrough infections (BTIs) influenced Ab responses, cross-neutralisation capacity, and B memory cell development. According to the clinical protocol, the primary objectives were the humoral immunogenicity to mRNA SARS-CoV-2 vaccination measured by ELISA and inhibition assay before and after the primary and three booster vaccinations. Secondary objectives were the cellular SARS-CoV-2 mRNA vaccine response with regard to B memory cells, the differences in vaccine responsiveness depending on MM disease stage and treatment, and the effects of occurring BTIs.

These immunological parameters should help to clarify whether and for which MM patients/subgroups individualised vaccination programmes are required.

We here demonstrate that repeatedly mRNA-vaccinated MM patients, particularly those receiving anti-CD38 mAb therapy, showed lower SARS-CoV-2 Wuhan- and Omicron-specific Ab responses and delayed S-specific B memory cell formation, which, however, improved in patients with hybrid immunity. Omicron SARS-CoV-2 infections were generally mild compared to previous SARS-CoV-2 strains but may also be due to functional cellular responsiveness in MM patients.

## 2. Methods

### 2.1. Study Design

The study was conducted at the Outpatient Vaccination Clinic at the Institute of Specific Prophylaxis and Tropical Medicine of the Medical University Vienna, Austria, where immune-compromised patients routinely receive care according to national vaccination guidelines. Patient-related study procedures and data collection, as well as laboratory analyses of the drawn samples, were conducted between March 2021 and December 2023. Written informed consent was obtained from all participants according to the Declaration of Helsinki/International Conference on Harmonization Guideline for Good Clinical Practice. The study was approved by the Ethics Committee of the Medical University Vienna (EK: 1073/2021), and international trial registration was completed at EudraCT, Reg. Number: 2021-000291-11.

### 2.2. Participants

Individuals who visited the Outpatient Vaccination Clinic as part of the Austrian COVID-19 immunisation campaign were invited to participate in this study. Adults aged ≥18 years with no previous SARS-CoV-2 exposure through infection or vaccination were included. Patients required a diagnosis of MGUS or MM with/without immunosuppressive/immunomodulatory therapy (IT). Healthy controls were excluded if they had any form of immunosuppression/-modulation (inclusion/exclusion criteria in Supplementary Data S1).

MM patients were stratified into five groups by disease stage and treatment and according to the Revised International Staging System (R-ISS) [9]: (1) MGUS, (2) MM patients after stem cell transplantation (SCT) without further therapy, (3) MM patients after SCT with IT (lenalidomid and/or pomalidomid and/or dexamethasone), (4) progressed MM patients, and (5) MM patients without SCT, IT, or any other treatment.

Participants with BTI (i.e., positive PCR and/or nucleocapsid protein-specific Abs in enzyme-linked immunosorbent assay [ELISA] during the observation period, Omicron wave starting in January 2021) were analysed separately and compared to uninfected participants.

The study sample consisted of 70 MM patients and 66 healthy controls. Of the enrolled participants, 47 MM patients and 38 controls received the complete two-dose SARS-CoV-2 mRNA primary vaccination (vaccine dose, vd1, vd2) and a booster (vd3) after six months. A fourth vaccination (vd4) was administered to the remaining 39 MM patients and 19 controls after six months, and26 MM patients and eight controls received a fifth vaccination (vd5) after another six months.

### 2.3. Procedures

**Figure 1:**
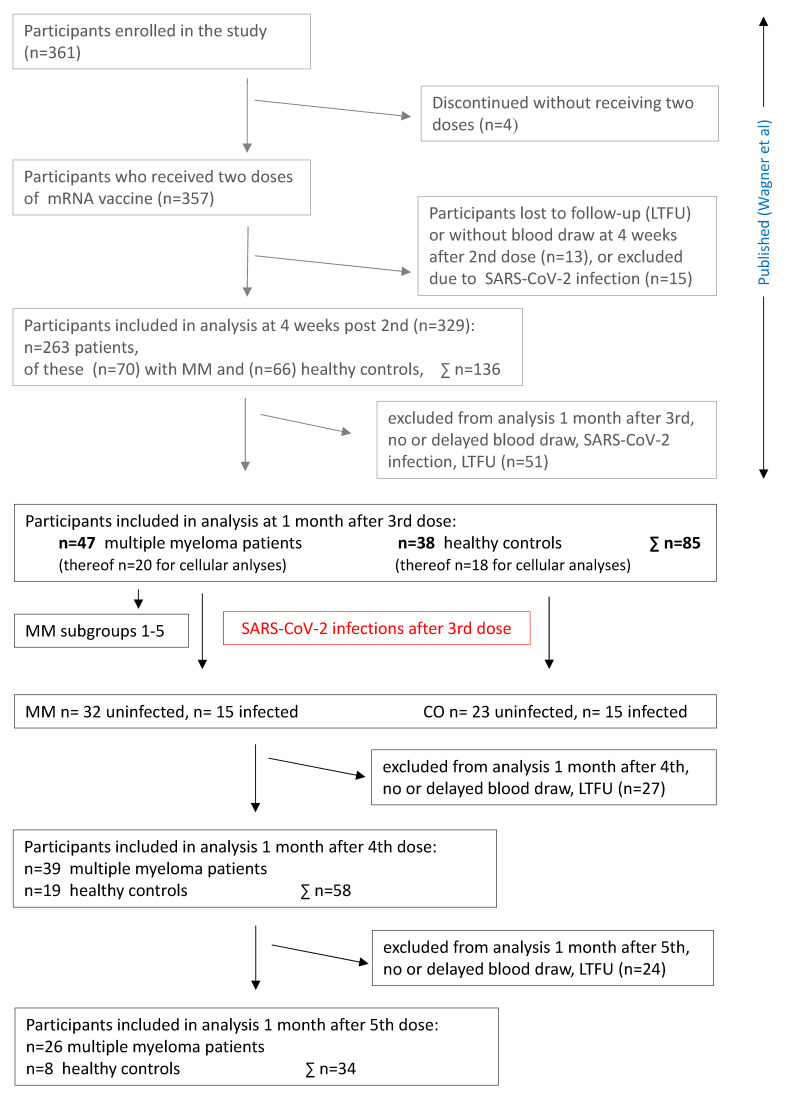
Flowchart. Abbreviations: MM, multiple myeloma; CO, control subjects; LTFU, lost to follow-up [4].

All participants received monovalent wildtype vaccine for the two-dose primary vaccination (and the third dose (booster) within the national COVID-19 program). Participants compliant with follow-up had a fourth and fifth vaccine dose, which could be monovalent or bivalent, i.e., wildtype or variant of concern (VOC)-adapted vaccines, depending on national vaccination guidelines and licensing status (Figure 1).

**Figure 2:**
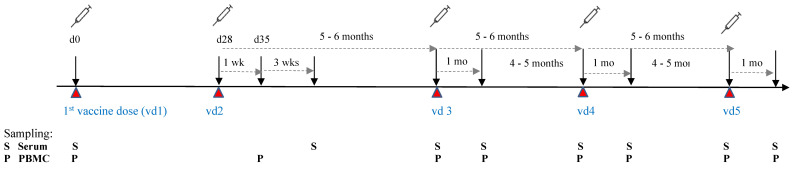
Timeline of Interventions. Abbreviations: d, day; mo, months; S, serum collection; P, PBMC collection; wk, week; vd, vaccine dose = time point of SARS-CoV-2 mRNA vaccination.

Blood samples for assessment of humoral and cellular vaccine responses were collected prior to the first dose and one and four weeks after the second dose, as well as prior to and four weeks after each booster dose (vd3, vd4, and vd5). Serum samples were obtained from native venous blood and peripheral blood mononuclear cells (PBMCs) were isolated from lithium-heparinised blood. Both were frozen and stored until analysis. PBMC isolation was carried out in a subset of participants (*n* = 20 MM patients, *n* = 18 controls) who consented to donation of the required additional blood volumes (Figure 2).

Lymphocyte subset distributions and SARS-CoV-2 S protein-specific B memory cells were analysed in MM patients with high versus low antibody responses. Ab high responders were defined as >2500 BAUs/mL one month after the second dose and >295 BAUs/mL after six months, and low responders as <1600 BAUs/mL one month and <295 BAUs/mL six months after the second dose.

### 2.4. SARS-CoV-2-Specific IgG Antibodies against Wuhan and Omicron Variants

SARS-CoV-2-specific IgG Abs directed against spike subunit 1 (S1) protein of the original strain (hu-1) were measured by ELISA (*Anti-SARS-CoV-2-QuantiVac-ELISA (IgG)*, Euroimmune^®^, Medizinische Labordiagnostik AG, Lübeck, Germany) in diluted serum samples (1:100) according to the manufacturer’s instructions. Ab results are reported in binding antibody units/mL (BAUs/mL) and were considered positive at ≥35.2 BAUs/mL. IgG specific for SARS-CoV-2 Omicron BA.4/5 and XBB.1.5 receptor-binding domain (RBD) were measured by ELISA (results as OD values, values > 0.25 were considered positive). The capacity to inhibit Omicron BA.4/5 and XBB.1.5 RBD binding to ACE2 (as % inhibition) was assessed as previously described, using 50 ng RBD BA.4/5 or XBB.1.5 and serum dilutions of 1:2; inhibition levels >20% were considered positive and >50% as clinically relevant [10].

### 2.5. Leukocyte and Lymphocyte Counts

Leukocytes and lymphocytes were measured in EDTA whole-blood samples with a SYSMEX XP-300 differential haematology analyser.

### 2.6. Cellular Immune Responses

*PBMC isolation*: PBMCs from lithium-heparinised blood were prepared by Ficoll density gradient centrifugation (Ficoll Paque Plus, GE Healthcare Europe GmbH, Freiburg Germany) and restimulated with S1-specific peptide pools from hu-1 at 0.03 nmol per peptide per 5 × 10^5^ cells (PepTivator^®^ SARS-CoV-2 peptide pools, Milteny Biotech, Bergisch Gladbach, Germany) for 24 h as previously described [4].

*Cytokine measurements*: Concentrations of interleukin (IL)-2 and interferon (IFN)-γ were measured in thawed supernatants with Luminex Human High-Sensitive Cytokine Performance Assays (Bio-Techne, Minneapolis, MN, USA), used according to the manufacturer’s instructions on a Luminex^®^ 100/200 System.

*Flow cytometric lymphocyte analysis*: PBMCs were surface stained with fluorochrome-conjugated monoclonal (m)Abs (Supplementary Data S2) to characterise B and T cell subsets. Data were acquired on a FACS Canto II flow cytometer by gating on cells with forward/side light scatter properties of lymphocytes and analysed with FACS Diva 8.0 software and FlowJo_v10.8.1 software (BD Biosciences, San Jose, CA, USA). Percentages of sub-populations related to the respective parent population and absolute numbers (n/µL) were calculated based on peripheral white blood cell counts. For the detection of SARS-CoV-2 S protein-specific B memory cells, biotinylated S protein (Wuhan, 1256 aa) antigen was tetramerised with streptavidin-APC or streptavidin-BV421 probes as described in Dan et al. [11] and S-specific memory B cells were quantified as percentages of total memory B cells as reported previously [8].

### 2.7. Statistical Methods

Antibody levels were expressed as BAUs/mL for S1-specific IgG and as OD values for BA.4/5- and XBB.1.5-specific IgG. These values showed a log-normal distribution and were analysed by choosing this distribution in the generalised estimating equations (GEE) model. Percent inhibition was logit transformed with 100% arbitrarily set to 99.5% and 0% to 0.5%. For the GEE model with unstructured correlation matrix, visit number was the within-subject variable and group (MM vs. controls) the between-subject factor of interest. For some analyses, MM patients were subdivided into four groups that were individually compared to controls. For each visit, dependent variables were compared between groups by linear contrasts with Sidak–Holm-corrected *p*-values. For all analyses, no imputations for missing values were applied and *p*-values below 0.05 were considered significant. Analyses were performed using Stata 17.0 (StataCorp, College Station, TX, USA), and graphs were produced using GraphPad Prism (San Diego, CA, USA; Version 9.3).

## 3. Results

### 3.1. Cohort Characteristics

There were 43% women among MM patients and 47% among controls. MM patients were a mean (SD) of 65.2 (9.1) years and controls 54.8 (13.8) years old, which was statistically significant (*p* < 0.001). The body mass index (BMI) in both groups was similar, although slightly more MM patients than controls were considered obese (BMI > 30 kg/m^2^). At enrolment, the mean (SD) time since MM diagnosis in affected patients was 7.6 (6) years (Table 1). All participants received monovalent wildtype (hu-1) vaccines for vd1, vd2, and vd3. For vd4 and vd5, an increasing proportion of subjects received Omicron-adapted vaccines (bivalent hu-1/Omicron BA.4/5 or monovalent Omicron XBB.1.5 vaccine, Supplementary Table S1). The discrepancy in use of vaccine type for MM patients and healthy controls is due to the fact that myeloma patients received boosters earlier according to national vaccination guidelines [12], which was prior to the licencing of bivalent vaccines. Importantly, the majority of the analysed uninfected controls received wildtype vaccine as vd4.

Of *n* = 47 MM patients, *n* = 7 had MGUS (group 1), *n* = 11 had received SCT without requiring further IT (group 2), *n* = 12 had had SCT and received IT (group 3), *n* = 12 had progressed MM (group 4), and *n* = 4 received no therapy (group 5). One patient could not be assigned to any group because even though he/she received no prior SCT, immunomodulatory treatment was administered, which is normally only prescribed to patients after SCT. Progressed MM patients were frequently treated with anti-CD38 mAb (daratumumab, as mono or part of double or triple therapy) or proteasome inhibitors (carfilzomib and/or bortezomib) (Table 2). Sufficient participant numbers allowed for analyses of subgroups (1) MGUS, (2) SCT without IT, (3) SCT with IT, and (4) progressed MM.

### 3.2. Reduced Ab Levels and Omicron Cross-Neutralisation in Vaccinated Uninfected MM Patients

**Figure 3:**
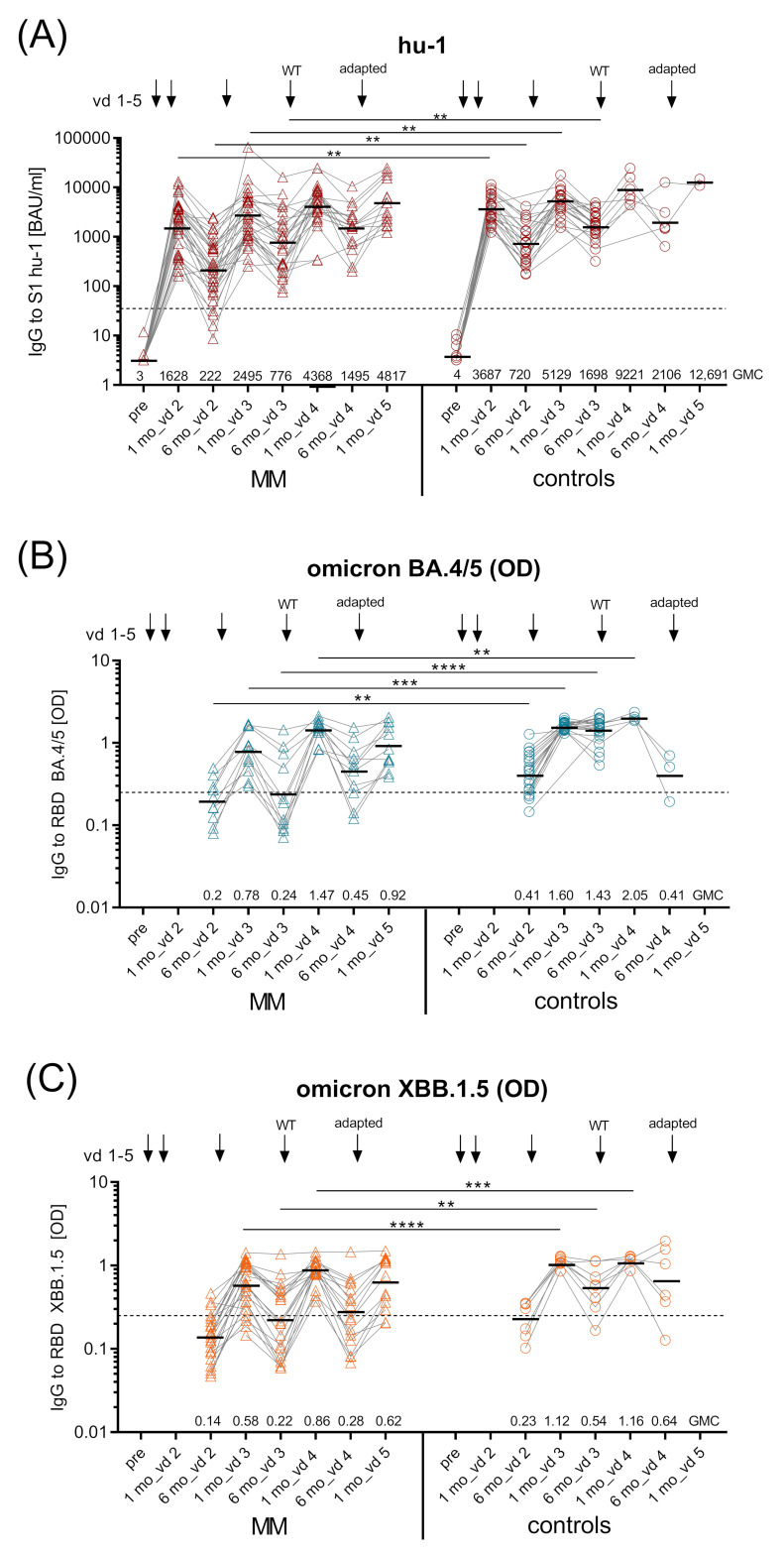

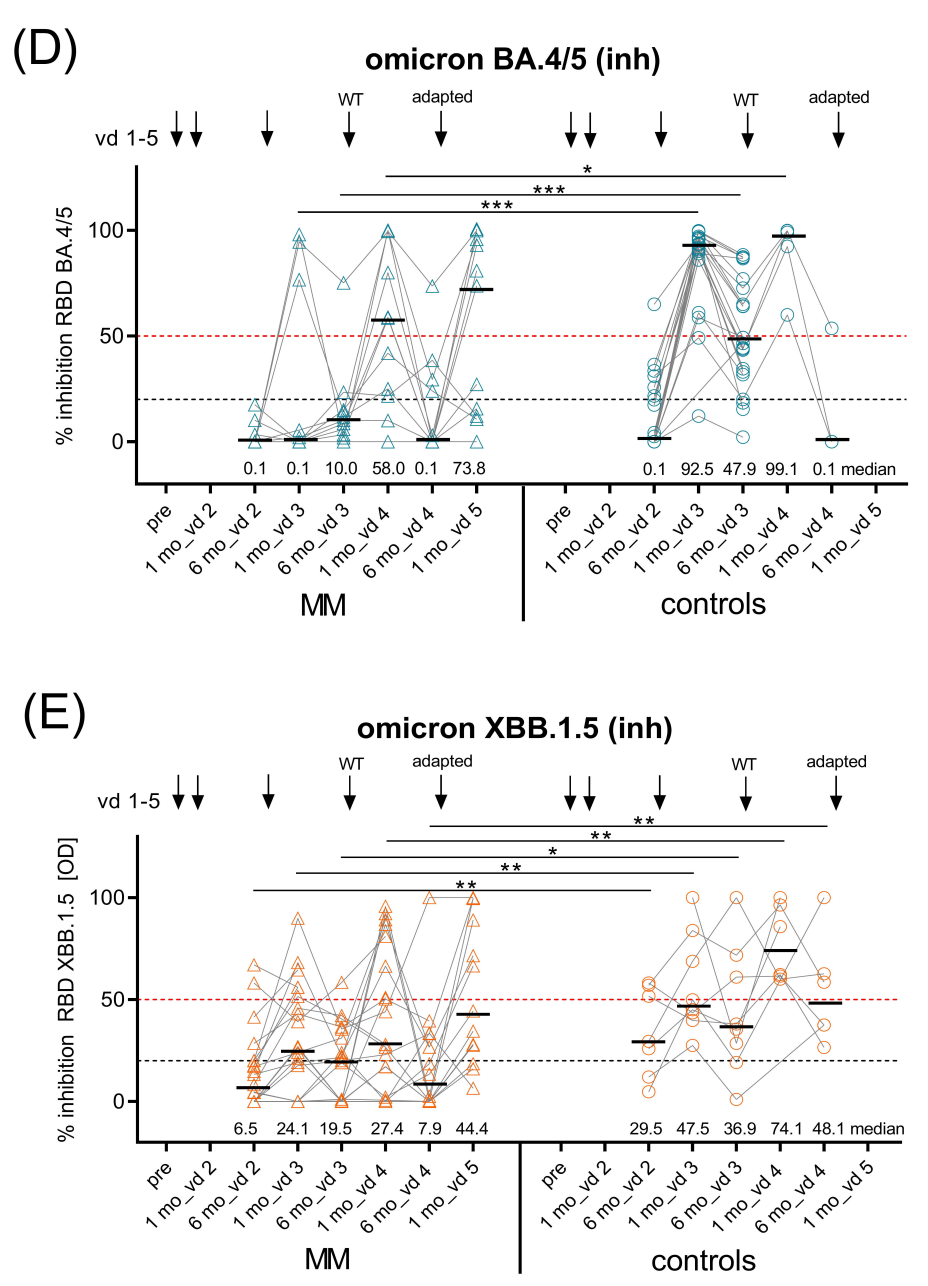
Kinetics of SARS-CoV-2 spike (S1)-specific IgG antibodies. Kinetics of GMC of (**A**) ancestral virus hu-1 S1-specific IgG (BAUs/mL) in uninfected MM patients (*n* = 32) and controls (*n* = 23) measured before vd1, one and six months after vd2, vd3, and vd4, and one month after vd5 of SARS-CoV-2 mRNA vaccine (BNT162b2 or mRNA-1273); dashed line—positive cut-off for S1-specific IgG at 35.2 BAUs/mL; (**B**) Omicron BA.4/5 RBD-specific IgG (as OD) in uninfected MM patients (*n* = 11) and controls (*n* = 20) six months after vd2, one and six months after vd3 and vd4, and one month after vd5 and C) Omicron XBB.1.5 RBD-specific IgG (as OD) in uninfected MM patients (*n* = 22) and controls (*n* = 7) at the same time points; black dashed lines in (**B**,**C**), OD > 0.25 considered positive, horizontal line indicates GMC provided numerically above x-axis; kinetics of inhibition capacity of (**D**) Omicron BA.4/5 RBD-specific IgG (as % inhibition) measured in uninfected MM patients (*n* = 11) and controls (*n* = 20) six months after vd2, one and six months after vd3 and vd4, and one month after vd5, and (**E**) Omicron XBB.1.5 RBD-specific IgG (as % inhibition) measured in uninfected MM patients (*n* = 22) and controls (*n* = 7) at the same time points; inhibition levels >20% considered positive (black dashed line), inhibition levels >50% relevant (red dashed line); horizontal line indicates median provided numerically above x-axis. Abbreviations: BAUs, binding antibody units; GMC, geometric mean concentrations; IgG, immunoglobulin G; mo, months; MM, multiple myeloma patients; mRNA, messenger ribonucleic acid; OD, optical density; RBD, receptor-binding domain; S1, SARS-CoV-2 spike protein 1; vd, vaccine dose. Linear contrasts with Sidak–Holm-corrected *p*-values; **** *p* ≤ 0.0001; *** *p* ≤ 0.001; ** *p* ≤ 0.01; * *p* ≤ 0.05.

SARS-CoV-2-specific IgG antibodies against hu-1 S1 protein were measured pre-vaccination and one and six months after the primary and each booster vaccination (Figure 2). One month after vd2, MM patients had mounted significantly lower geometric mean Ab concentrations (GMC 1624 BAUs/mL (95% CI 1060–2486)) than healthy controls (GMC 3687 BAUs/mL (95% CI 2813–4833), *p* < 0.01). Six months thereafter, S1-specific IgG had declined to significantly lower levels in MM patients compared to controls (GMC 224 BAUs/mL (95% CI 134–374) vs. GMC 720 BAUs/mL (95% CI 475–1091), *p* < 0.01), with eight percent of MM becoming seronegative. Importantly, vd3 increased S1-specific IgG in both groups; however, levels after one month remained significantly lower in MM patients than in controls (GMC 2502 BAUs/mL (95% CI 1664–3763) vs. GMC 5229 BAUs/mL (95% CI 3940–6677), *p* < 0.01), and similarly so after six months (*p* < 0.01). After vd4, Ab levels in patients and controls were no longer significantly different, which might be attributed to the low number of controls receiving vd4 (Figure 3A).

In addition to hu-1 S1-specific IgG, we also measured IgG specific for the receptor-binding domain (RBD) of the Omicron variants BA.4/5 and XBB.1.5. The GMCs of Omicron BA.4/5 RBD-specific Abs were significantly lower in MM patients six months after vd2 (optical density, OD 0.2 vs. 0.43, *p* < 0.01) and one month after vd3 (OD 0.79 vs. 1.6, *p* < 0.001) compared to controls, while Omicron XBB.1.5 RBD IgG were reduced only one month after vd3 (OD 0.58 vs. 1.12, *p* < 0.0001) (Figure 3B,C). The decline in VOC-specific IgG at six months after vd3 was much more prominent in MM patients than in controls, because the majority of MM patients (63% for Omicron BA.4/5 and 55% for XBB.1.5) but only one control vaccinee showed Ab levels considered negative at this time point. One month after vd4, all MM patients had positive levels of VOC-specific IgG yet GMCs were still lower than in controls (*p* < 0.001).

Furthermore, we assessed cross-neutralisation of the vaccine-induced Abs in MM patients and controls, i.e., capacity to inhibit Omicron BA.4/5 or XBB.1.5 RBD binding to the ACE-2 receptor. The potential to inhibit Omicron BA.4/5 RBD binding was greatly reduced in MM patients at one and six months after vd3 (both *p* < 0.001) compared to controls. This was the case also after vd4 (*p* < 0.05), where, at analysis, the majority of MM patients and controls had received the Wuhan vaccine. The capacity to cross-inhibit Omicron XBB.1.5 RBD binding was significantly lower than in controls at all timepoints (Figure 3D,E).

### 3.3. Degree of Vaccine Failure in Uninfected MM Patients Depends on Disease Stage and Treatment

**Figure 4:**
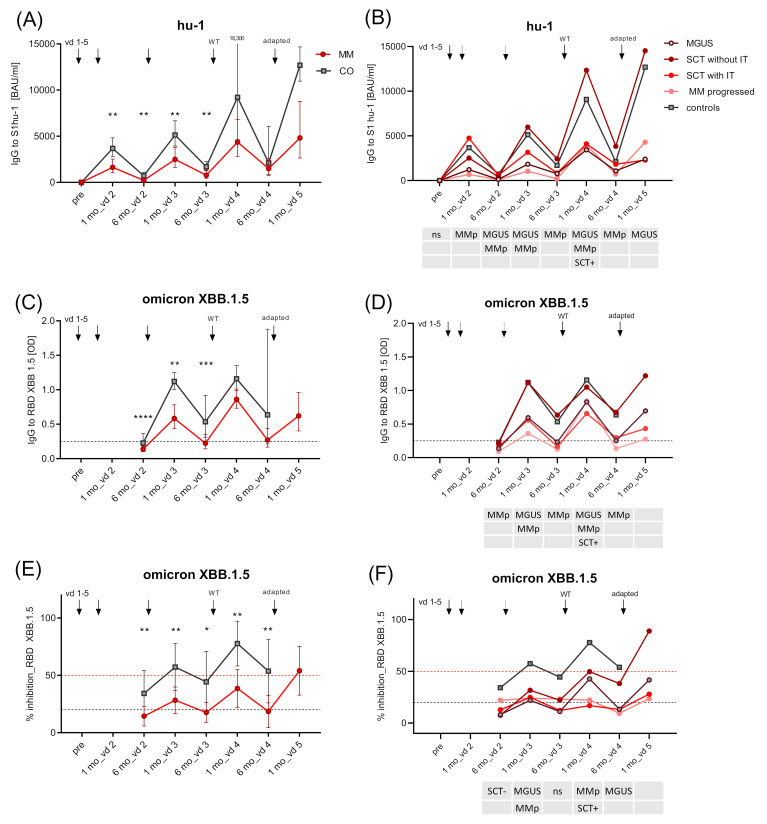
Kinetics of SARS-CoV-2-specific IgG Ab levels (hu-1 and XBB.1.5) in entire MM group and MM subgroups. Kinetics of SARS-CoV-2 Wuhan S1-specific IgG (BAUs/mL) (**A**) for controls and entire MM group as GMC with 95% CI; (**B**) for controls and MM subgroups as GMC (95% CI provided in Supplementary Table S2A); kinetics of SARS-CoV-2 Omicron XBB.1.5 RBD-specific IgG (OD) for (**C**) controls and entire MM group as GMC with 95% CI; (**D**) for controls and MM subgroups as GMC (95% CI provided in Supplementary Table S2B); kinetics of SARS-CoV-2 Omicron XBB.1.5 RBD-binding (as % inhibition) for (**E**) controls and entire MM group as mean with 95% CI, and (**F**) for controls and MM subgroups as mean (95% CI provided in Supplementary Table S2C). MM subgroups: MGUS (dark red line), MM after SCT and no further immunomodulatory treatment (red line), MM after SCT with immunomodulatory treatment (light red line), progressed MM (pink line), and healthy controls (grey line) before vd1, one and six months after vd2, vd3, and vd4, and one month after vd5 of SARS-CoV-2 mRNA vaccine; for OD graphs: dashed black line—OD values >0.25 considered positive; for inhibition graphs: levels >20% considered positive (black dashed line), >50% relevant (red dashed line). Abbreviations: BAUs, binding antibody units; CI, confidence interval; GMC, geometric mean concentration; mo, months; OD, optical density; RBD, receptor-binding domain; S1, SARS-CoV-2 spike protein 1; SCT, stem cell transplant; SD, standard deviation; vd, vaccine dose. The table below the *x*-axis shows the subgroups that are significantly (*p* < 0.05) different from controls at the respective time point (linear contrast with Sidak–Holm-corrected *p*-values). *p*-values; **** *p* ≤ 0.0001; *** *p* ≤ 0.001; ** *p* ≤ 0.01; * *p* ≤ 0.05.

Ab kinetics were assessed in four MM patient subgroups: (1) MGUS, (2) SCT without further immune-modulating therapy, (3) SCT with IT, and (4) progressed MM patients; (5) MM without therapy was too small for analysis (*n* = 4). Group 2 (SCT without IT) responded equally well as controls with respect to hu-1-S1 and XBB.1.5 RBD-specific Ab levels. In contrast, patients with MGUS (group 1), after SCT with IT (group 3), and progressed MM patients (group 4) mounted significantly lower Ab levels compared to controls (Figure 4B,D, Supplementary Table S2A,B).

Cross-neutralising capacity varied between groups. Omicron XBB.1.5 RBD inhibiting capacity was below 50% in all MM groups and controls until six months after vd3; controls reached inhibition > 50% one months after vd4 and group 2 (SCT without IT) one month after vd5 (Figure 4F, Supplementary Table S2C). Data for Omicron BA.4/5 IgG levels and inhibition capacity show that responsiveness for group 2 (SCT without IT), though of low sample size, was similar to that of controls (Supplementary Figure S1A–F).

### 3.4. Breakthrough Infections Increase Ab Levels and Omicron-Cross Protection in Vaccinated MM Patients

**Figure 5:**
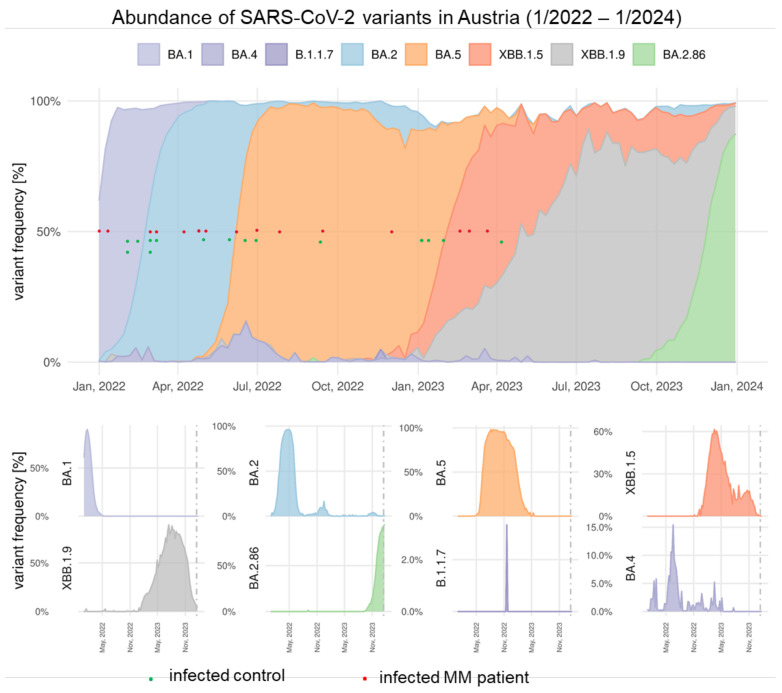
Time of breakthrough infections (BTIs) in MM and controls in relation to occurrence of SARS-CoV-2 variants. Juxtaposition of the time of BTI and the relative abundance of circulating SARS-CoV-2 at that time, as deduced through the Austrian National SARS-CoV-2 Wastewater Monitoring, for which the influent of 48 wastewater treatment plants, serving ~60% of the Austrian population, are sampled. Relative abundance of variants of concern, following Pango nomenclature, is deduced by tiling whole-genome sequencing and variant deconvolution performed by the software tool VaQuERo [13]. Red dots indicate timepoints of BTIs in MM patients; green dots indicate time points of BTIs in control subjects.

BTIs began to occur in MM patients (*n* = 15) and controls (*n* = 15) at some point between one and six months after vd3. The infecting strains were not identified in the individual patients by sample sequencing, but time of infection and relative abundance of strains (according to surveillance by waste water analyses) [13] suggests BTIs with Omicron BA.1*, BA.2*, BA.5*, and XBB.1.5* variants (Figure 5).

**Figure 6:**
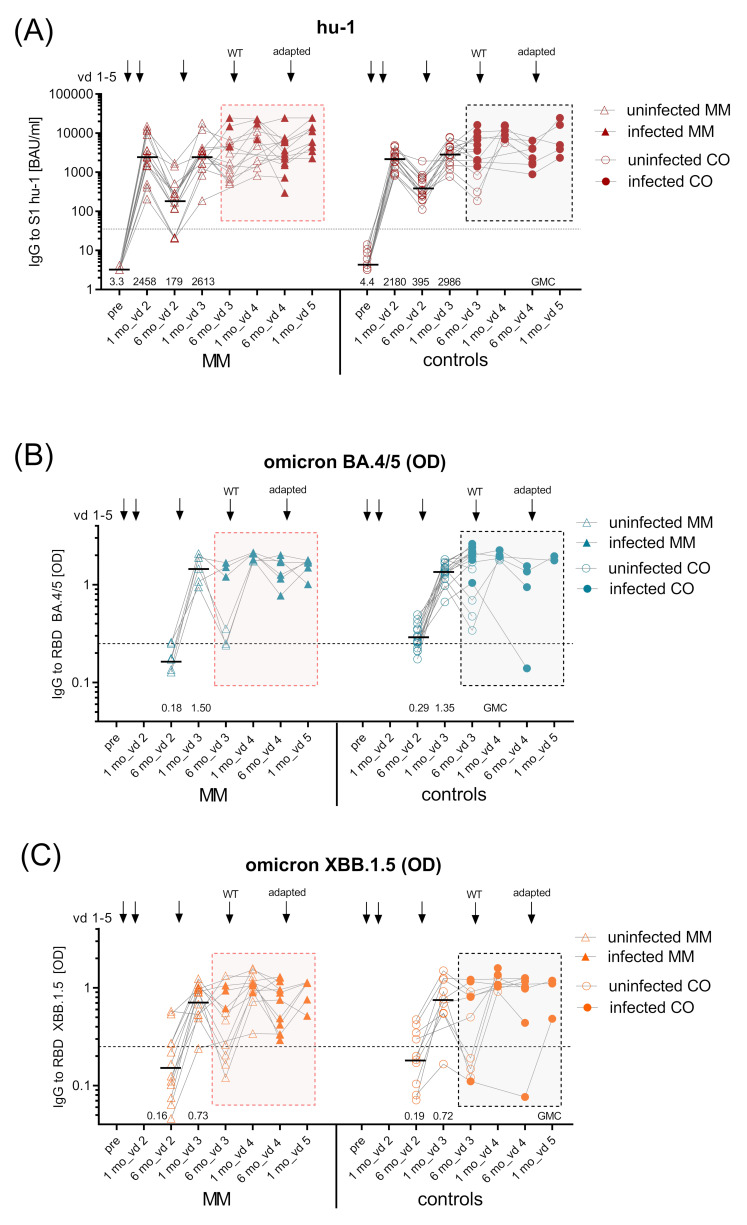

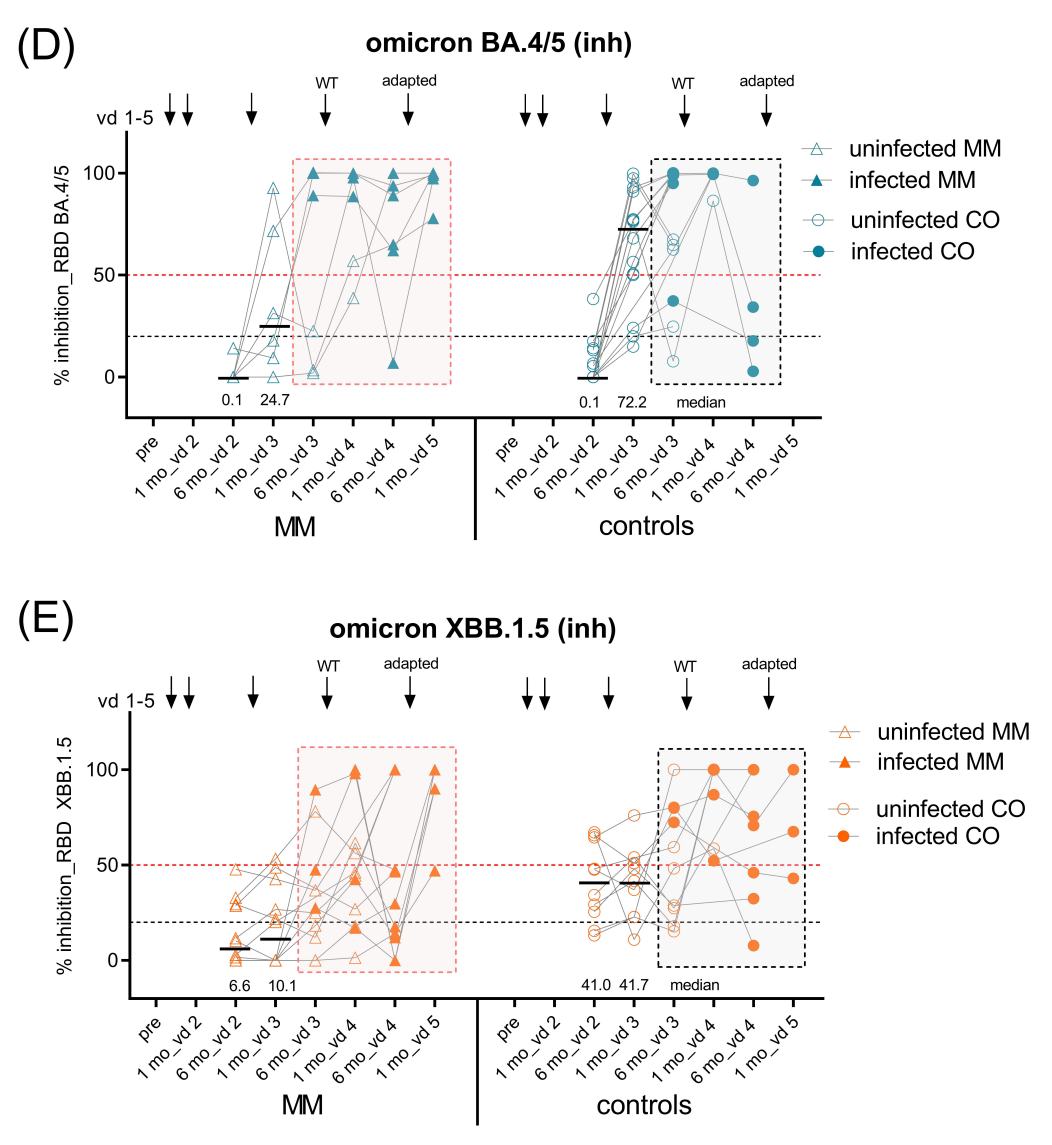
Kinetics of SARS-CoV-2 hu-1, Omicron BA.4/5- and XBB.1.5-specific Abs in MM and controls with breakthrough infections. Kinetics of (**A**) ancestral virus hu-1 S1-specific IgG in BAUs/mL in infected MM patients (*n* = 15) and infected controls (*n* = 15) measured before vd1, one and six months after vd2, vd3, and vd4, and one month after vd5 of SARS-CoV-2 mRNA vaccine (BNT162b2 or mRNA-1273); dashed line—positive cut-off for S1-specific IgG at 35.2 BAUs/mL; (**B**) Omicron BA.4/5 RBD-specific IgG (as OD) in infected MM patients (*n* = 6) and infected controls (*n* = 15) six months after vd2, one and six months after vd3 and vd4, and one month after vd5; (**C**) Omicron XBB.1.5 RBD-specific IgG in infected MM patients (*n* = 12) and infected controls (*n* = 10) (as OD) at the same timepoints; black dashed lines in B and C, OD > 0.25 considered positive, horizontal line indicates GMC provided numerically above x-axis; kinetics of inhibition capacity (as % inhibition) of (**D**) Omicron BA.4/5 RBD-specific IgG in infected MM patients (*n* = 6) and infected controls (*n* = 15) six months after vd2, one and six months after vd3 and vd4, and one month after vd5; and (**E**) Omicron XBB.1.5-RBD-specific IgG in infected MM patients (*n* = 12) and infected controls (*n* = 10) at the same timepoints; inhibition levels >20% considered positive (black dashed line), inhibition levels >50% relevant (red dashed line), horizontal line indicates median provided numerically above x-axis; uninfected—empty symbols, infected—full symbols. Abbreviations: BAUs, binding antibody units; CO, control subjects, GMC, geometric mean concentration; IgG, immunoglobulin G; MM, multiple myeloma patients; mo, months; mRNA, messenger ribonucleic acid; OD, optical density; RBD, receptor-binding domain; S1, SARS-CoV-2 spike protein 1; vd, vaccine dose.

BTIs occurred in all groups, independent of MM disease stage and treatment type; furthermore, all infected individuals presented with relatively mild COVID-19 disease courses (no hospitalisation or death). BTIs not only inhibited Ab waning—as seen in uninfected vaccinated subjects—but also increased Ab responses (Figure 6A, full symbols). BTIs after vd3 also induced high levels of Omicron BA.4/5- and XBB.1.5 RBD-specific Abs in MM patients and controls up to six months after vd3. Booster vaccinations (vd4) following BTIs could not further elevate antibody levels (Figure 6B,C; full symbols).

Of note, cross-neutralisation to VOCs (i.e., inhibition of Omicron BA.4/5 and XBB.1.5 RBD binding to ACE-2) was strongly increased after BTI in MM patients and controls (Figure 6D,E); full symbols. 

Individuals with BTI/hybrid immunity (MM patients and controls) showed significantly higher hu-1 S1-specific IgG compared to their vaccinated uninfected counterparts at six months after vd3. Application of vd4 did not further increase Ab levels in infected subjects but did so in uninfected subjects (Supplementary Figure S2A). The same comparison of VOC-specific Abs showed that, in particular, BA.4/5 Abs (Supplementary Figure S2B) and their inhibition capacity (Supplementary Figure S2D) were significantly elevated in infected vs. uninfected vaccinated individuals six months after vd3. With the application of vd4, however, uninfected MM patients and controls reached similar levels of Omicron BA.4/5-specific Ab as infected, but the BA.4/5 cross-neutralisation remained lower in uninfected patients than in those with hybrid immunity (Supplementary Figure S2B–D).

### 3.5. Lymphocyte Distributions and SARS-CoV-2 S-Protein-Specific Memory B Cells in MM Ab High and Low Responders

We investigated to which degree MM treatment with SCT and/or different biological immuno-modulators influenced the baseline distributions of lymphocyte subsets in myeloma patients. The patients who were considered high responders* (*n* = 8) were mainly those after SCT with and without IT, while low responders** (*n* = 12) were mostly progressed patients receiving anti-CD38 mAb treatment (daratumumab) as monotherapy or as part of double or triple therapy (Table 2). *Ab high responders were defined as >2500 BAUs/mL one month after second dose and >295 BAUs/mL after six months, and **low responders as <1600 BAUs/mL one month and <295 BAUs/mL six months after second dose.

#### 3.5.1. Leukocyte and Lymphocyte Counts

Mean leukocyte numbers were similar in MM high and low responders (mean 5.05 × 10^3^/µL (95% CI 3.73–6.36) vs. 5.28 × 10^3^/µL (95% CI 4.30–6.18), respectively). Lymphocytes percentages (of leukocytes) and absolute numbers (n/µL) were both slightly increased in high vs. low responders ((31.2% lymphocytes (95% CI 23.0–39.5) vs. 25.6% (95% CI 20.4–30.8); 1.54 lymphocytes × 10^3/^µL (95% CI 0.99–2.08) vs. 1.33 × 10^3^/µL (95% CI 0.99–1.67), respectively) (Supplementary Figure S3A–C).

#### 3.5.2. CD3+ T Cells, CD19+ B Cells, and NK T Cells

Mean levels of CD3+ T cells and CD19+ B cells as percentages of total lymphocytes and in absolute numbers (n/µL) were normal. Percentages of total CD3+ T cells were significantly increased in low responders (CD3+ T cells 75.7% (95% CI 66.6–84.7) vs. 54.4% (95% CI 44.2–64.7)), yet not as absolute counts (Supplementary Figure S4A,B). Percentages of the CD4+ and CD8+ T cell subset were by trend increased in low responders, while NK T cells were significantly increased both as percentages and absolute numbers (Supplementary Figure S4C–H). In contrast to T cells, CD19+ B cells as relative percentages and absolute numbers were significantly reduced in low responders (CD19+B cells 6.3% (95% CI 3.3–9.3) vs. 11.5% (95% CI 7.6–15.4); absolute B cells 0.89 × 10^3^/µL (95% CI 0.45–1.33) vs. 1.84 × 10^3^/µL (95% CI 0.91–2.78), respectively) (Supplementary Figure S5A,B). In low responders, immature transitional B cells (CD19+/CD24^high^/CD38^high^) and plasmablasts (PBs) (CD19+/CD27++/CD38^high^), in particular as absolute counts, were significantly reduced (Supplementary Figure S5C–F). Furthermore, NK cells calculated as percentages of lymphocytes (%NK = 100% lymphocytes – (%CD3+ T cells + %CD19+ B cells)) and as absolute numbers (n/µL) (Supplementary Figure S6A,B) were also diminished in low responders.

#### 3.5.3. SARS-CoV-2 Spike (S) Protein-Specific Memory B Cells

**Figure 7:**
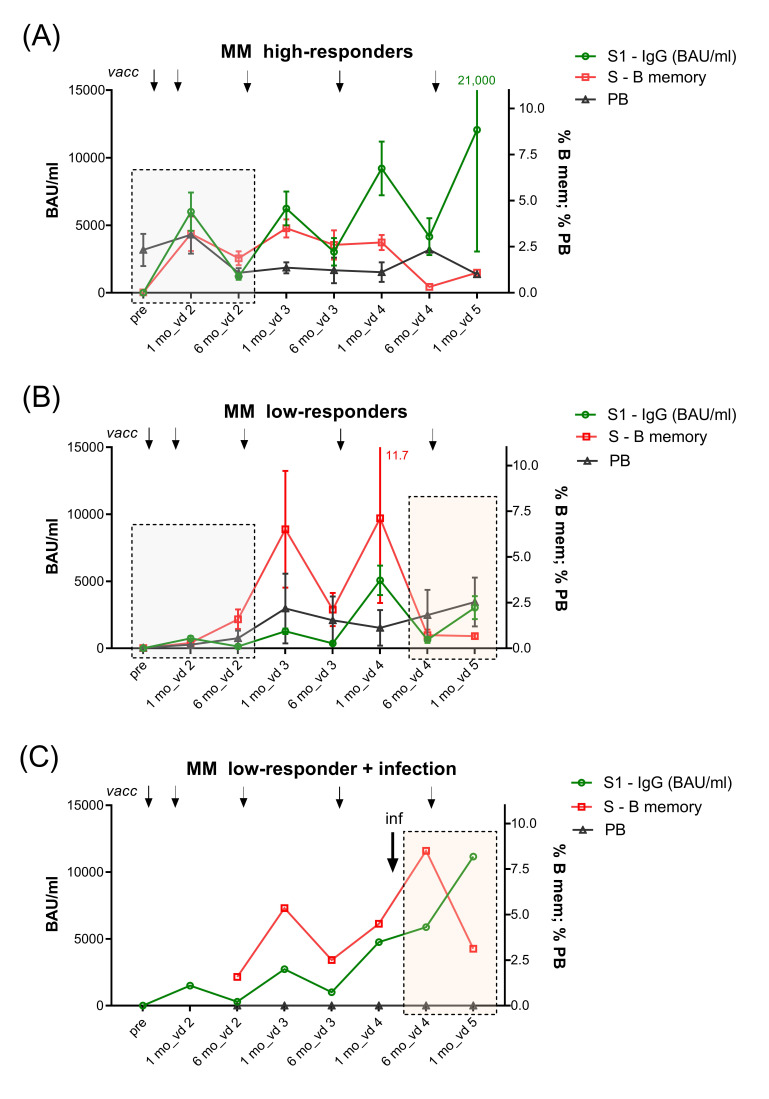
Kinetics of S1-specific IgG, S-specific memory B cells, and plasmablasts in high- and low-responder MM vaccinees. Kinetic of S1-specific IgG (in BAUs/mL), S-protein-specific memory B cells (as % of total memory B cells), and plasmablasts (PBs; as percentages of total CD19+B cells) determined before vd1 and either one week (S-specific memory B and PBs) or one month (S1-specific IgG) after vd2, one and six months after vd3 and vd4, and one month after vd5 of SARS-CoV-2 mRNA vaccine in (**A**) MM Ab high responders (*n* = 8; S1-specific IgG >2500 BAUs/mL one month after vd2 and >295 BAUs/mL six months after vd2) and (**B**) MM Ab low responders (*n* = 10; S1-specific IgG < 1600 BAUs/mL one month after vd2 and <295 BAUs/mL six months after vd2), data points represent arithmetic mean with SEM, and (**C**) in one exemplary MM low-responder patient with breakthrough infection in the time period between one and six months after vd4. Grey boxes in A and B indicate the difference in B memory cell responses between high- and low-responders; rose-coloured boxes in B and C indicate differences in B memory cell and Ab kinetics in MM low responders without and with infection. Abbreviations: Ab, antibody; BAUs, binding antibody units; IgG, immunoglobulin G; inf, infection; MM, multiple myeloma; PBs, plasmablasts; RBD, receptor-binding domain; S1, SARS-CoV-2 spike protein 1; SEM, standard error of the mean; vd, vaccine dose.

Similar to controls (Supplementary Figure S7), MM high responders (*n* = 8) formed high numbers of S-specific memory B cells one week after vd2. These S-specific memory B cells remained stable or further expanded until six months after vd2 and further increased after vd3 (Figure 7A). In contrast, low responders (*n* = 12) showed delayed formation of S-specific memory B cells, reaching detectable levels only six months after vd2, which further increased after vd3 (Figure 7B). Concerning further boosters, vd4 led to the production of high antibody levels in MM high-responders but no longer the formation of peripheral S-specific memory B cells. This was in contrast to low responders, where vd4 triggered production of both Abs and S-specific memory B cells and only vd5 led to an increase in Abs but not memory B cells (Figure 7A,B). Regarding the influence of infection on S-specific memory B cell development in MM low responders, we observed that BTI between vd4 and vd5 counteracted the expected decline and resulted in an increase in both Ab levels and S-specific memory B cells, as exemplified in one MM low responder in Figure 7C.

### 3.6. SARS-CoV-2-Specific Cytokines Are Induced in MM Patients after Third and Fourth Vaccine Dose

We analysed T cell responses, i.e., concentrations of cytokines IFN-γ and IL-2 in re-stimulated PBMC culture supernatants at the time points before and one month after vd3 and vd4 in uninfected vaccinees. After primary vaccination, IL-2 but not IFN-γ levels had correlated with S1-specific IgG in MM patients [4]. After the first booster (vd3), levels of both IL-2 and IFN-γ correlated with S1-specific IgG, and at the time of BTI (between vd3 and vd4), when antibodies had already declined in MM patients, IFN-γ levels were still present in considerable levels, and this remained so up to one month after vd4 (Supplementary Figure S8A–H).

## 4. Discussion

This work describes the follow-up of SARS-CoV-2 mRNA vaccine responses in MM patients over three years, who received three booster doses (vd3, vd4, and vd5) with monovalent hu-1 (vd3, vd4) and/or Omicron-adapted vaccines (vd5). We and others have shown that immune responses to primary COVID-19 mRNA vaccination are insufficient in myeloma patients [4,6]. Our results here confirm existing data after booster vaccination [14], as we show that vd3 and vd4 increased the levels of hu-1-specific Abs, but they still remained below the levels of healthy controls (Figure 3A). Vd3 of the ancestral strain failed to induce long-lasting Omicron-specific Ab levels (BA.4/5 and XBB.1.5 RBD), and cross-neutralisation capacity was greatly reduced (Figure 3B–E), similar to what was demonstrated by others [15,16]. We here evaluated the effects of further boosters along with breakthrough infections/hybrid immunity in treatment-differentiated MM subgroups.

Depending on disease stage and treatment, we observed great variations in levels and functionality of vaccine-induced Abs. As we and others have described for primary vaccination [4,5,6], responses to vd3 and vd4 with monovalent vaccine were inferior in some MM groups compared to healthy individuals: Patients with MGUS, progressed MM stage with anti-CD38 mAb, and MM after SCT with IT mounted low and fast-waning IgG levels against hu-1 and Omicron strains without significant neutralisation capacity against VOCs. Omicron-adapted vd5 moderately increased cross-protection in patients with MGUS and MM after SCT without IT, but not in MM after SCT with IT or progressed MM patients receiving anti-CD38 mAb (Figure 4D,F), similar to reports by Aleman et al. [17]. These results support our previous observation in IBD patients [8], namely, that detailed subgroup analysis is crucial to identifying patients, who will remain vulnerable to existing and emerging VOCs despite several booster vaccinations.

In particular, MM patients receiving anti-CD38 remain at risk for infection. This is explainable by the fact that CD38, the type II transmembrane glycoprotein to which the mAb binds, is expressed on plasma cells, plasmablasts, and immature transitional B cells, and are highly present on malignant myeloma cells [18]. According to the function of anti-CD38, MM Ab low-responders had depleted plasmablasts and immature transitional B cells, resulting in reduced total B cell numbers and in turn increased CD3+ T cells (Supplementary Figures S4 and S5) [17,18,19,20]. We demonstrated here that these deficits were associated with a delayed formation of S-specific memory B cells after two-dose primary vaccination. Moreover, MM low responders showed a parallel increase in S-specific memory B cells and IgG up to vd4, and vd5 increased only Abs without further expansion of memory B cells (Figure 7B). In contrast, MM Ab high responders and healthy controls concomitantly developed S-specific memory B cells and IgG only until after vd3, and from then on boosters increased Ab levels without B cell expansion (Figure 7A and Supplementary Figure S7). This finding supports that immune cell-depleting therapies inhibit the timely formation of antigen-specific memory B cell pools. Accordingly, in-vitro data have demonstrated that anti-CD38 mAb impairs switched memory B cell development [21], and Aleman et al. reported that delayed formation of memory B cells was due to missing interactions between depleted dendritic cells, T-follicular helper cells, and B cells [17]. The delay of memory B cell responses in MM patients is in contrast to the underlying mechanisms of defective memory B cell maintenance in anti-TNF-α-treated IBD patients, which is caused by base-line inflammation and high pro-inflammatory cytokines [8].

Large retrospective studies in vaccinated cancer patients have shown low SARS-CoV-2 infection rates but still increased hospitalisation and deaths in those who became infected [22]. With respect to MM, only those patients with chemo- or immunotherapy experienced significantly more BTIs with more severe clinical outcome [23]. Also, in our cohort participants became infected, but myeloma patients showed neither more frequent infections nor more severe disease than controls. BTIs started to occur between one and six months after vd3 and were followed by stable hu-1- and VOC-specific Ab levels compared to declining Abs in uninfected vaccinated MM patients and controls (Figure 6A–C). Importantly, hybrid immunity improved cross-neutralisation to Omicron variants, in particular in MM patients (Supplementary Figure S2D,E). Of interest is that BTI also led to an expansion of S-specific memory B cells, which might improve long-term cross-protection (Figure 7C). Hybrid immunity is described to be of greater magnitude and durability and has improved protective effectiveness against Omicron variants than immunity following vaccination only [24,25]. Our findings show that this also accounts for immunocompromised MM patients. The mild disease courses in myeloma patients could firstly be due to a rather unaffected T cell compartment. We previously showed diminished T cell responses after primary vaccination in these patients [4], as also reported by others [26]. However, after booster vaccination—similarly to what is described elsewhere [16]—we observed that MM Ab low responders had considerable IFN-γ levels at the time of BTI (Supplementary Figure S8G), indicating a certain protective capacity. In addition, these patients had expanded CD8+ cytotoxic T cells (Supplementary Figure S4E,F), which were shown to prevent severity and mortality of COVID-19 in MM patients. [27]. The protective role of T cells after BTI was demonstrated by others in myeloma and B cell lymphoma patients [28,29], and chronic lymphocytic leukaemia patients with hybrid-immunity had highly functional T cells with cross-recognition of Omicron spike epitopes [30]. Secondly, infections with the Omicron variant in general caused less severe disease at the population level [31], and thirdly, MM patients are classified as a risk population, and therefore, some received anti-viral treatment upon infection.

In addition to current expert consensus statements in the post-pandemic COVID-19 aera [32,33], our results provide data elucidating the effects of BTIs and help in the guidance of MM patient management. The strength of our study is that we longitudinally evaluated vaccine responses in an MM cohort according to different treatment groups and at different stages of immune competence. Another benefit is that we separately analysed/compared vaccinated patients with and without BTI, which so far has not been evaluated by others. A possible limitation might be the rather small sample size; however, even with these limited cohorts, we identified statistically significant differences, and thus, the reported results seem meaningful and sound. The age difference between the control and MM groups might be considered a potential limitation. However, our previous study on aging immunity and vaccination by Wagner et al. [34] showed significant differences in vaccine responses only in cohorts with a large age difference of 45 years (mean 24 vs. 69 y). Furthermore, 45% of the control subjects were at an age (i.e., >60 y) where immune--senescence is already established. Thus, while we cannot exclude that the higher mean age of the MM group did influence vaccination outcomes, the impact might not have been so profound as to explain the significant response difference between the MM group and healthy controls.

In summary, we have shown that MM patients benefited from SARS-CoV-2 booster vaccinations regarding Ab levels but not cross-neutralisation capacity against VOCs, in particular in patients with progressed myeloma. In this group, a B cell-depleted phenotype led to delayed generation of memory B cells. Furthermore, BTIs in MM patients improved cross-neutralisation against VOCs without causing severe COVID-19. We conclude that myeloma patients will remain susceptible to newly emerging viral strains and should be encouraged to receive variant-specific boosters, and that a change in vaccine platform [35,36] could further broaden their nAb repertoire (similar to what was seen after BTI). However, these efforts should not only focus on SARS-CoV-2 but also on other vaccine-preventable diseases, where responsiveness might be impacted by treatment in a similar manner.

## Figures and Tables

**Table 1:** Cohort characteristics.

	*p*-Value	MM (n = 47)	Controls (n = 38)
Gender (female), *n* (%)	0.668	20 (43%)	18 (47%)
Age (years), mean (SD)	<0.001	65.2 (9.1)	54.8 (13.8)
Age < 60 years, *n* (%)		12 (25.5)	21 (55.2)
BMI (kg/m^2^), mean (SD)	0.599	25.9 (4.3)	25.4 (4.4)
BMI > 30, *n* (%)	0.601	12 (26%)	7 (18%)
Time since diagnosis (years), mean (SD)		7.6 (6)	n. a.

Abbreviations: BMI, body mass index; MM, multiple myeloma; n. a., not applicable; SD, standard deviation.

**Table 2:** Description of MM subgroups.

	MGUS	SCT w/o IT	SCT with IT *	MM Progressed	No SCT, IT or Other Therapy	Total *n*
Group number	G 1	G 2	G 3	G 4	G 5	
						
*n* per group	7	11	12	12	4	46
SCT	0	11	12	10	0	33
>2 y since SCT at inclusion		9	8	6		23
>2 y since SCT at 6 mo after vd3		10	10	10		30

Treatment in G4 (MM Progressed)	No SCT + dara mono	SCT + dara or + dara & dexa	SCT + dara (of double or triple)	No SCT + PI	SCT + PI	
*n* per group	1	5	4	1	1	12

Abbreviations: dara, Daratumumab; dexa, Dexamethasone; IT, immunomodulatory treatment; MGUS, monoclonal gammopathy of undetermined significance; MM, multiple myeloma; mo, months; n. a., not applicable; PI, proteasome inhibitor; SCT, stem cell transplant;vd, vaccine dose; y, years. * IT: lenalidomid and/or pomalidomid and/or dexamethasone.

## Data Availability

Ethics approval does not allow data sharing of person-related data. Upon reasonable request and depending on a positive ethics vote, raw data supporting the conclusions of this article can be made available by the principal investigator.

## Supplementary Materials

The following supporting information can be downloaded at: https://www.mdpi.com/article/10.3390/vaccines12050518/s1, Figure S1: Omicron BA.4/5 RBD-specific IgG Ab levels (OD) and RBD-binding to ACE2 (as % inhibition), Figure S2: Comparison of hu-1 S1, BA.4/5, and XBB.1.5 specific IgG Ab levels in infected vs. uninfected controls and MM patients, Figure S3: Leukocyte and lymphocyte counts pre-vaccination, Figure S4: Quantification of T lymphocytes and the CD4, CD8 and NK-T-cell subset pre-vaccination, Figure S5: Quantification of B lymphocytes, immature transitional B cells and plasmablasts pre-vaccination, Figure S6: Quantification of NK cell subset pre-vaccination, Figure S7: Kinetics of S1-specific IgG, S-specific memory B cells and plasmablasts in healthy controls, Figure S8: Correlations of-hu-1 S1 specific IgG Abs with concentrations of cytokines IFN-γ and IL-2 in PBMC culture supernatants, Table S1: Booster vaccinations in MM and Controls, Table S2: Statistics for hu-1 S1 and XBB.1.5 specific IgG in MM subgroups, Data S1: Inclusion and Exclusion criteria, Data S2: Fluorochrome-conjugated monoclonal Abs for flow-cytometric analysis of B and T cell panel.
