# Supplementary material for: Breakthrough Infections in SARS-CoV-2-Vaccinated Multiple Myeloma Patients Improve Cross-Protection against Omicron Variants
Source: Vaccines (Basel). 2024 May 9;12(5):518. doi: 10.3390/vaccines12050518 (PMC11125692; doi:10.3390/vaccines12050518)
Supplement: Supplementary file 1 [file vaccines-12-00518-s001.zip › vaccines-2961181-supplementary.pdf]

Supplementary Material

Table of contents:

Supplementary Table S1: *Booster vaccinations in MM and Controls* *page 2*

Supplementary Table S2: *Statistics for hu-1 S1 and Omicron XBB.1.5 specific IgG in MM subgroups* *page 3*

Supplementary Figure S1: *Omicron BA.4/5 RBD-specific IgG Ab levels (OD) and RBD-binding to ACE2 (as % inhibition)* *page 4-5*

Supplementary Figure S2: *Comparison of hu-1 S1, BA.4/5, and XBB.1.5 specific IgG Ab levels in infected vs. uninfected controls and MM patients* *page 6-8*

Supplementary Figure S3: *Leukocyte and lymphocyte counts pre-vaccination* *page 9*

Supplementary Figure S4: *Quantification of T lymphocytes and the CD4, CD8 and NK-T-cell subset pre-vaccination* *page 10*

Supplementary Figure S5: *Quantification of B lymphocytes, immature transitional B cells and plasmablasts pre-vaccination* *page 11*

Supplementary Figure S6: *Quantification of NK cells pre-vaccination* *page 12*

Supplementary Figure S7: *Kinetics of S1-specific IgG, S-specific memory B cells and plasmablasts in healthy controls* *page 13*

Supplementary Figure S8: *Correlations of-hu-1 S1 specific IgG Abs with concentrations of cytokines IFN-γ and IL-2 in PBMC culture supernatants* *page 14*

Supplementary Data S1: *Inclusion and exclusion criteria* *page 15*

Supplementary Data S2: *Fluorochrome-conjugated monoclonal Abs for flow-cytometric analysis* *page 15*

Supplementary Table S1: *Booster vaccinations in MM and Controls*

	MM (n=47)	Controls (n=38)
**Third dose (vd3)**	**47 (100)**	**38 (100)**
Wildtype	47 (100)	38 (100)
**Fourth dose (vd4)**	**39 (83)**	**19 (50)**
Wildtype	37/39 (95)	10/19 (53)
Wildtype and Omicron BA.1	0	1/19 (5)
Wildtype and Omicron BA.4/5	2/39 (5)	7/19 (37)
Omicron XBB.1.5	0	1/19 (5)
**Fifth dose (vd5)**	**26 (55)**	**8 (21)**
Wildtype	3/26 (11)	0
Wildtype and Omicron BA.4/5	21/26 (81)	4/8 (50)
Omicron XBB.1.5	2/26 (8)	4/8 (50)

Abbreviations: MM, multiple myeloma; vd, vaccine dose

Supplementary Table S2: *Statistics for hu-1 S1 and XBB.1.5 specific IgG in MM subgroups*

A)

**IgG to S1 hu-1 (BAU/mL)**	**MGUS (1)**			**SCT without IT (2)**			**SCT with IT (3)**			**MM progressed (4)**			**controls**		
	n=	GMC	95% CI	n=	GMC	95% CI	n=	GMC	95% CI	n=	GMC	95% CI	n=	GMC	95% CI
pre	4	3	3-3	8	3	3-3	6	3	3-4	10	3	3.2-3.2	23	4	3-4
1 mo_2nd	4	1223	161 -9315	8	2522	1265-5026	6	4740	1800-12479	9	685	249.6-1882	23	3687	2813-4833
6 mo_2nd	4	136	15-1207	8	406	162-1016	6	600	192-1872	8	100	32.5-305.4	21	720	476-1091
1 mo_3rd	4	1835	918-3667	8	5983	2082-17197	6	3173	1288-7820	9	1041	568.3-1906	21	5129	3940-6677
6 mo_3rd	4	763	114-5095	8	2437	727-8167	5	835	188-3722	7	205	107.9-388.2	23	1698	1279-2253
1 mo_4th	4	3438	1350-8754	5	12342	5542-27482	3	4106	1363-12372	7	3927	1350-11428	7	9080	5222-15788
6 mo_4th	4	1084	175-6722	5	3827	1542-9494	2	1834	106-31623	5	741	249.9-2197	6	2106	730-6079
1 mo_5th	3	2397	1043-5506	4	14531	4430-47657	1	2294	..	4	4297	837-22060	2	12691	1992-80849

B)

**IgG to RBD Omicron XBB.1.5 (OD)**	**MGUS (1)**			**SCT without IT (2)**			**SCT with IT (3)**			**MM progressed (4)**			**controls**		
	n=	GMC	95% CI	n=	GMC	95% CI	n=	GMC	95% CI	n=	GMC	95% CI	n=	GMC	95% CI
6 mo_2nd	4	0.14	0.05-0.39	6	0.21	0.12-0.39	4	0.17	0.07-0.39	7	0.09	0.05-0.17	7	0.23	0.14-0.37
1 mo_3rd	4	0.60	0.27-1.31	6	1.08	0.94-1.34	4	0.56	0.15-2.08	8	0.36	0.22-0.60	8	1.12	1.00-1.25
6 mo_3rd	4	0.23	0.05-1.14	6	0.55	0.34-1.17	3	0.17	0.01-2.07	7	0.13	0.06-0.25	8	0.54	0.31-0.92
1 mo_4th	4	0.83	0.47-1.48	6	1.07	0.78-1.40	3	0.66	0.27-1.588	7	0.84	0.58-1.21	6	1.16	0.99-1.35
6 mo_4th	4	0.25	0.05-1.18	5	0.77	0.26-1.78	2	0.30	0.00-342.80	5	0,14	0,06-0,30	6	0,64	0,21-1,88
1 mo_5th	3	0.70	0.15-3.25	4	1.22	0.97-1.53	1	0.43	..	4	0,27	0,15-0,50	0	..	..

C)

**% inhibition of Omicron XBB.1.5.**	**MGUS (1)**			**SCT without IT (2)**			**SCT with IT (3)**			**MM progressed (4)**			**controls**		
	n=	mean	95% CI	n=	mean	95% CI	n=	mean	95% CI	n=	mean	95% CI	n=	mean	95% CI
6 mo_2nd	4	8.1	-3.2-19.4	5	7.5	-5.3-20.2	4	12.8	-7.8-33.4	7	22.0	-4.7-48.6	7	34.2	14.0-54.3
1 mo_3rd	4	21.9	-29.4-73.1	5	31.6	-10.9-74.1	4	24.8	-21.4-71.0	8	24.1	2.2-46.0	8	57.3	36.7-77.9
6 mo_3rd	4	11.1	-9.3-31.3	5	22.2	5.0-39.3	3	12.2	-40.3-64.7	7	23.1	0.9-45.3	8	44.4	18.0-70.9
1 mo_4th	4	42.6	-22.3-107.6	5	49.7	-0.7-100.0	3	16.9	-55.1-88.8	7	22.3	-4.2-48.7	6	77.7	58.2-97.2
6 mo_4th	4	13.4	-11.2-37.9	4	38.1	-30.9-107.1	3	13.2	-43.5-69.8	5	9.5	-8.7-27.7	6	53.8	26.0-81.6
1 mo_5th	3	41.6	-26.1-109.2	4	88.8	63.9-113.7	1	28.0	..	4	23.6	-2.3-49.6	0	..	..

Abbreviations: GMC, geometric mean concentration; IT, immunomodulatory treatment; MGUS, monoclonal gammopathy of undetermined significance; MM, multiple myeloma; mo, months; n. a., not applicable; SCT, stem cell transplant; y, years

Supplementary Figure S1: *Omicron BA.4/5 RBD-specific IgG Ab levels (OD) and RBD-binding to ACE2 (as % inhibition)*

Depiction of A) Omicron BA.4/5 RBD-specific IgG (OD) as **GMC with 95% CI** for controls and entire MM group, B) Omicron BA.4/5 RBD-specific IgG (OD) as **GMC with 95% CI provided below** (S1E) for controls and MM subgroups, C) Omicron BA.4/5 RBD-binding to ACE2 (as % inhibition) as **mean with 95 % CI** for controls and entire MM group, and D) Omicron BA.4/5 RBD-specific to ACE2 (as % inhibition) as mean **with 95% CI provided below** (S1F) for controls and MM subgroups.

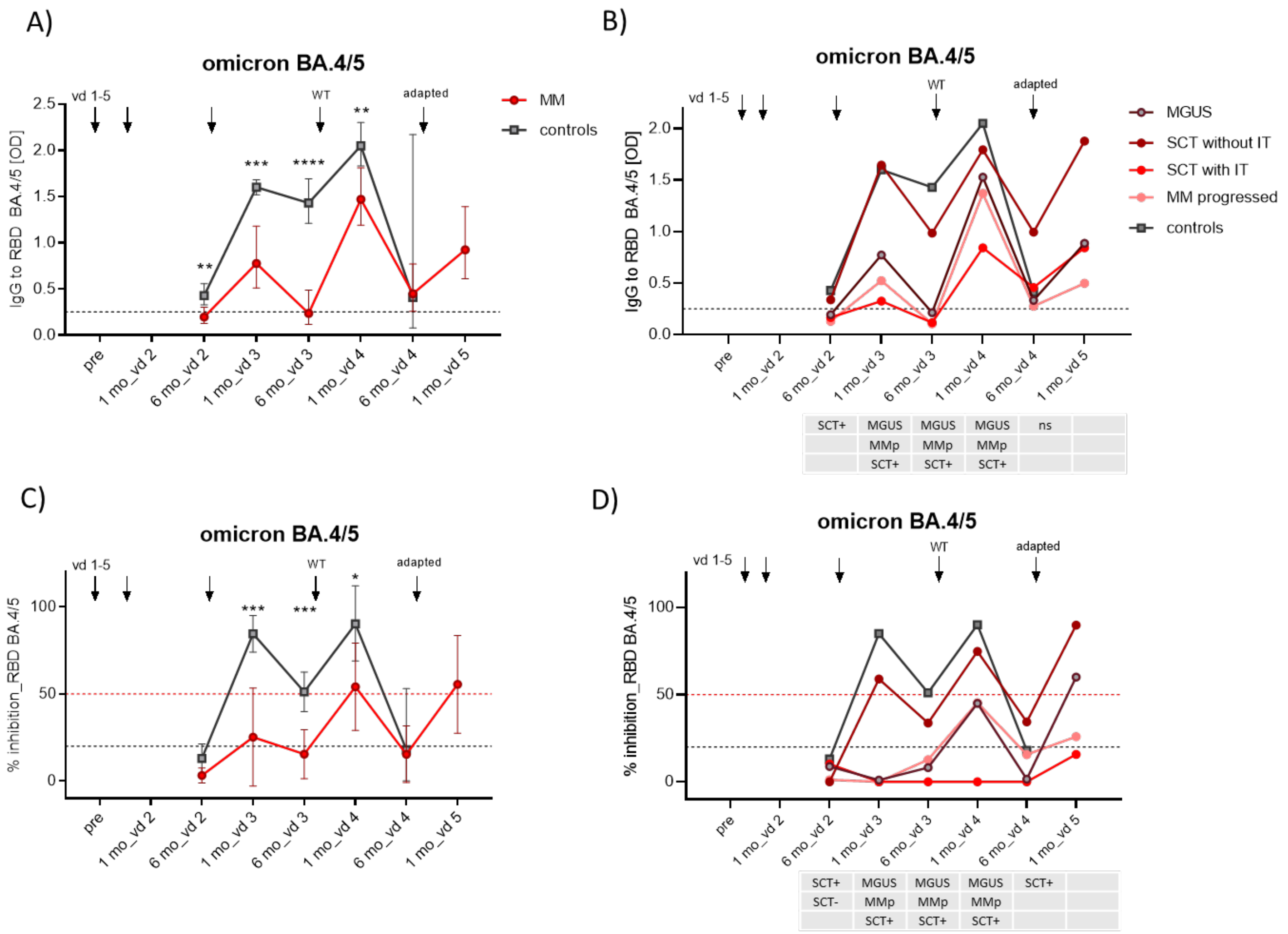

E)

IgG to RBD Omicron BA.4/5 (OD)	MGUS (1)			SCT without IT (2)			SCT with IT (3)			MM progressed (4)			controls		
	n=	GMC	95% CI	n=	GMC	95% CI	n=	GMC	95% CI	n=	GMC	95% CI	n=	GMC	95% CI
6 mo_2nd	2	0.19	0.00-2292	3	0.34	0.15-0.77	1	0.17	..	3	0.13	0.04-0.46	20	0.43	0.33-0.56
1 mo_3rd	2	0.77	0.06-9.47	3	1.65	1.54-1.76	1	0.33	..	4	0.52	0.25-1.09	20	1.60	1.52-1.68
6 mo_3rd	2	0.21	0.00-9775	3	0.99	0.42-2.30	1	0.12	..	4	0.10	0.05-0.20	22	1.43	1.21-1.69
1 mo_4th	2	1.53	0.31-7,57	3	1.79	1.19-2.72	1	0.84	..	4	1.37	0.77-2.43	5	2.05	1.83-2.30
6 mo_4th	2	0.33	0.00-17850	3	0.99	0.26-3.76	1	0.46	..	4	0.28	0.09-0.83	3	0.41	0.08-2.17
1 mo_5th	2	0.89	0.01-91.93	3	1.88	1.56-2.26	1	0.84	..	4	0.50	0.34-0.73	0	..	..

F)

% inhibition of Omicron BA.4/5	MGUS (1)			SCT without IT (2)			SCT with IT (3)			MM progressed (4)			controls		
	n=	mean	95% CI	n=	mean	95% CI	n=	mean	95% CI	n=	mean	95% CI	n=	mean	95% CI
-6 mo_2nd	2	8.8	-102.0-120.0	3	0.0	0.0-0.0	1	10.2	..	3	1.1	-3.7-6.0	20	13.0	4.4-21.0
1 mo_3rd	2	1.0	-12.0-14.0	3	58.9	-57.9-175.8	1	0.0	..	4	0.0	-0.0-0.0	20	85.0	74.0-95.0
6 mo_3rd	2	8.1	-19.0-35.0	3	33.7	-55.7-123.1	1	0.0	..	4	12.7	-1.1-26.4	22	51.0	40.0-63.0
1 mo_4th	2	45.0	-399.0-489.0	3	74.7	-32.3-181.7	1	0.0	..	4	45.3	17.3-73.3	5	90.0	69.0-112.0
6 mo_4th	2	1.5	-18.0-21.0	3	34.4	-57.8-126.5	1	0.0	..	4	15.6	-14.6-45.8	3	18.0	-59.0-95.0
1 mo_5th	2	60.0	-359.0-479.0	3	89.8	55.0-124.7	1	15.7	..	4	26.0	-33.0-84.9	0	..	..

Supplementary Figure S2: *Comparison of hu-1 S1, BA.4/5, and XBB.1.5 specific IgG Ab levels in infected vs. uninfected controls and MM patients*

Comparison of A) *SARS-CoV-2* S1-specific IgG against ancestral virus hu-1 in BAU/mL in uninfected and infected MM patients and controls measured six months after vd2, one and six months after vd3, one and six months after vd4 and one month after vd5 of SARS-CoV-2 mRNA vaccine (BNT162b2 or mRNA-1273); dashed line - positive cut-off for S1-specific IgG at 35.2 BAU/mL, B) Omicron BA.4/5 RBD-specific IgG and C) Omicron XBB.1.5 RBD-specific IgG (as OD, positive cut-off at OD 0.25) at the same timepoints; for A-C depiction of GMC & 95 % CI. Comparison of inhibition capacity of D) Omicron BA.4/.5 RBD-specific Abs, and E) Omicron XBB.1.5 RBD-specific Abs (as % inhibition) at the same timepoints, for D and E depiction of mean with SEM; inhibition levels of >20% were considered positive (black dashed line), inhibition levels >50% as relevant (red dashed line).

Abbreviations: BTI, break through infection, BAU, binding antibody units; Co, controls; IgG, immunoglobulin G; mo, months; mRNA, messenger ribonucleic acid; MM, multiple myeloma; OD, optical density; RBD, receptor-binding domain; S1, SARS-CoV-2 spike protein 1; vd, vaccine dose.

Mann-Whitney signed rank test for six months after vd2 and one months after vd3, Kruskal Wallis for further comparisons; ****p ≤0.0001; ***p ≤0.001; **p ≤0.01; *p ≤0.05

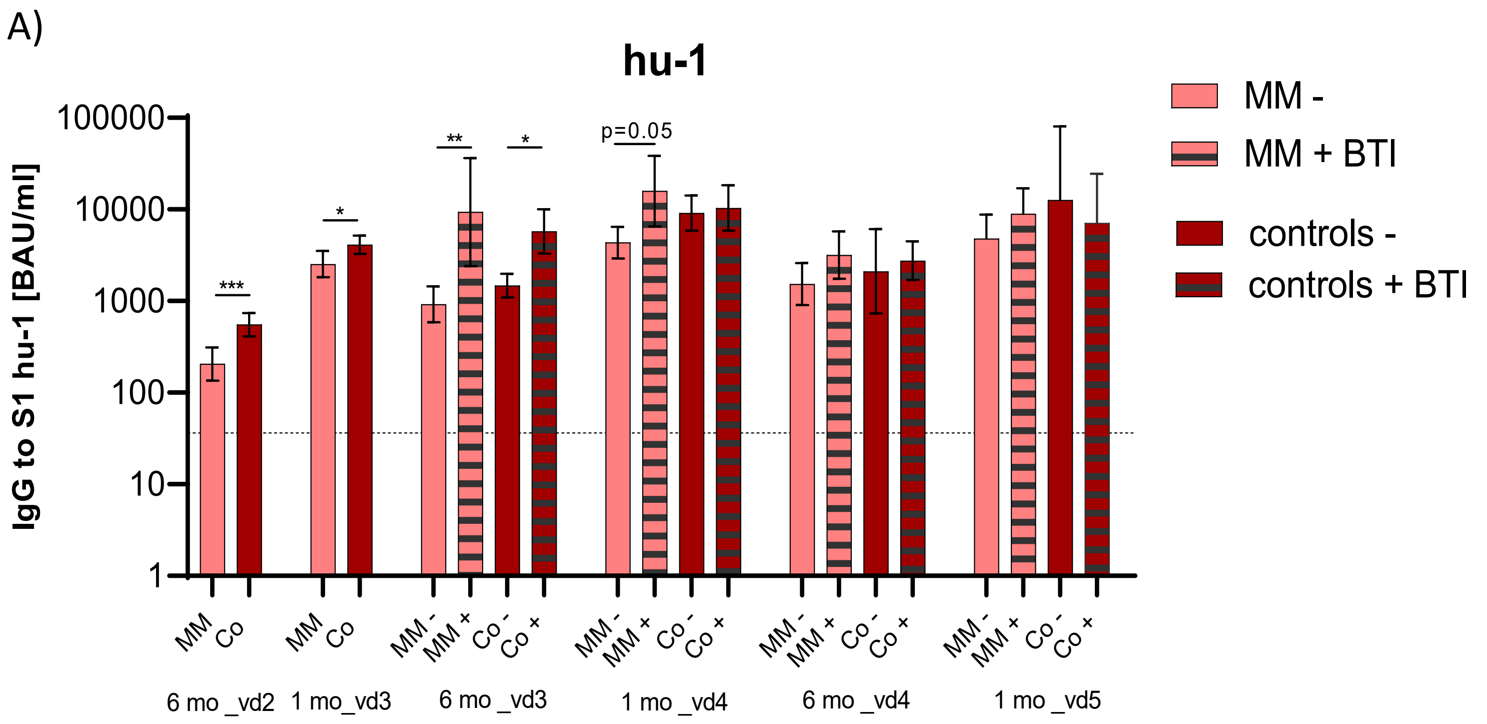

B)

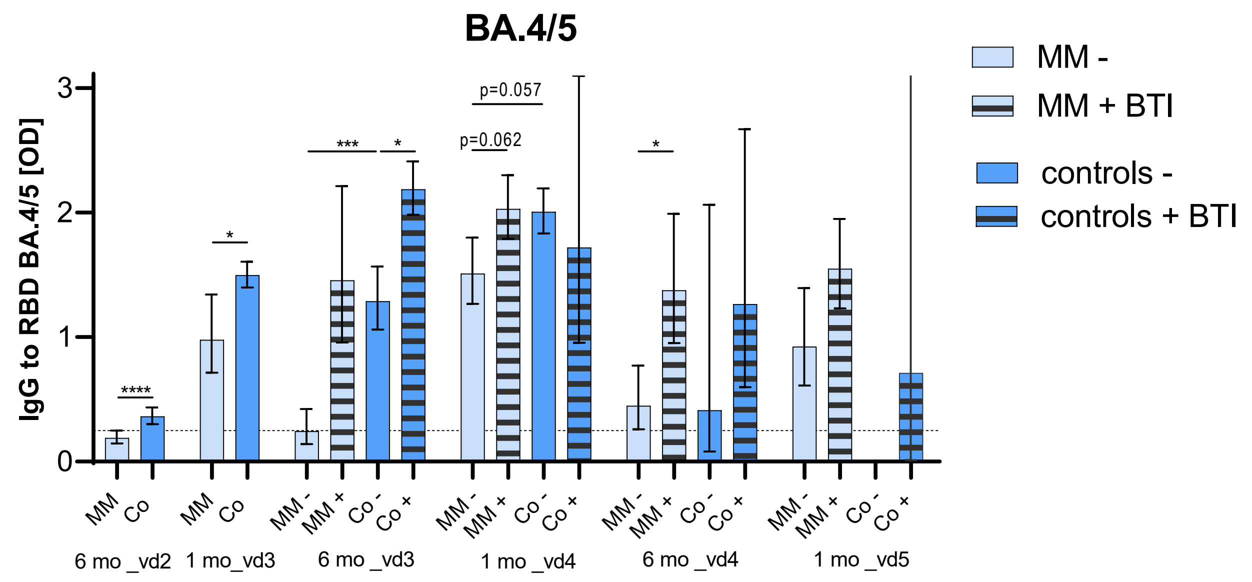
BA.4/5
IgG to RBD BA.4/5 [OD]
0
1
2
3
****
*
***
*
p=0.057
p=0.062
*
MM
Co
MM -
MM +
Co -
Co +
6 mo _vd2
1 mo_vd3
6 mo _vd3
1 mo _vd4
6 mo _vd4
1 mo _vd5
MM -
MM + BTI
controls -
controls + BTI

C)

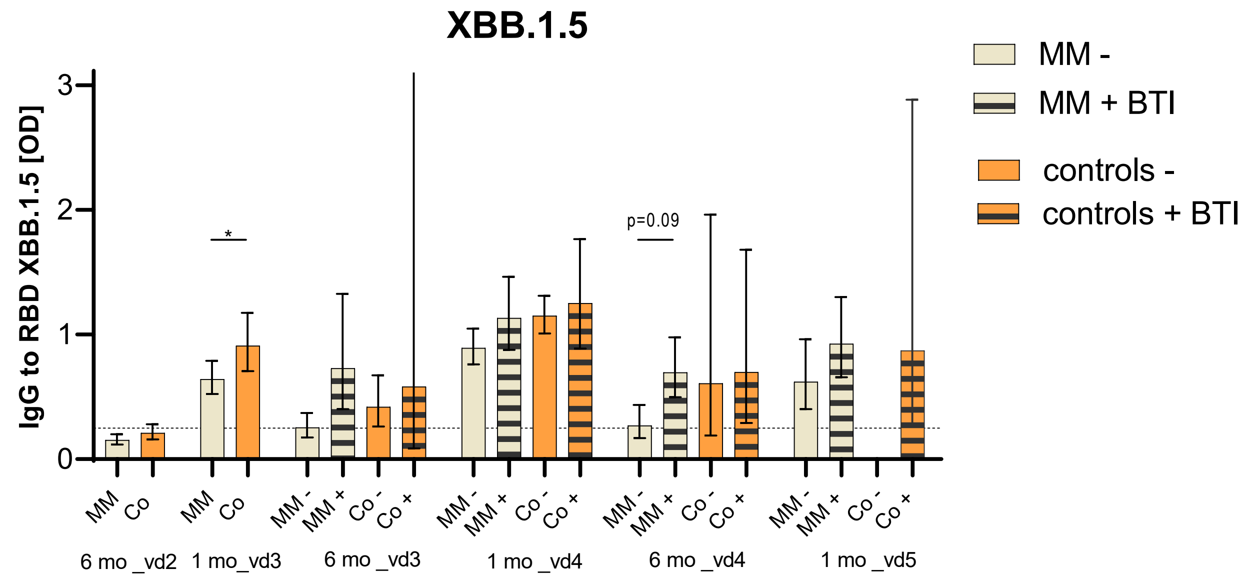
XBB.1.5
IgG to RBD XBB.1.5 [OD]
0
1
2
3
*
p=0.09
MM
Co
MM -
MM +
Co -
Co +
6 mo _vd2
1 mo_vd3
6 mo _vd3
1 mo _vd4
6 mo _vd4
1 mo _vd5
MM -
MM + BTI
controls -
controls + BTI

D)

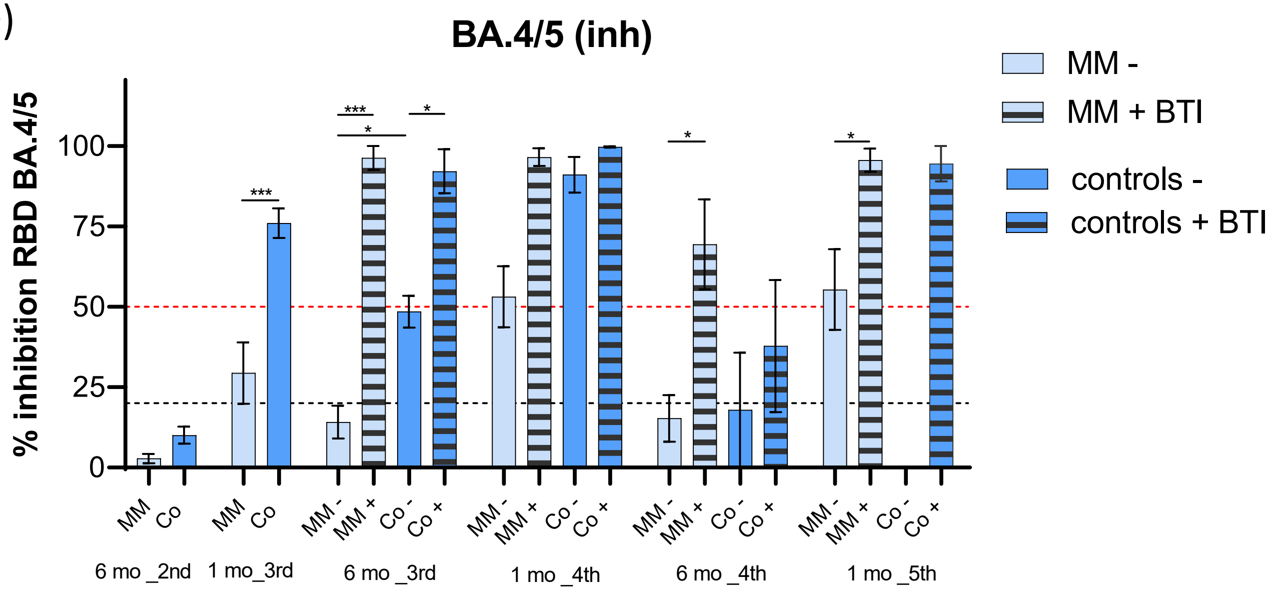
BA.4/5 (inh)
% inhibition RBD BA.4/5
0
25
50
75
100
***
***
*
*
*
*
MM
Co
MM
Co
MM -
MM +
Co -
Co +
MM -
MM +
Co -
Co +
MM -
MM +
Co -
Co +
MM -
MM +
Co -
Co +
6 mo _2nd
1 mo_3rd
6 mo _3rd
1 mo _4th
6 mo _4th
1 mo _5th
MM -
MM + BTI
controls -
controls + BTI

E)

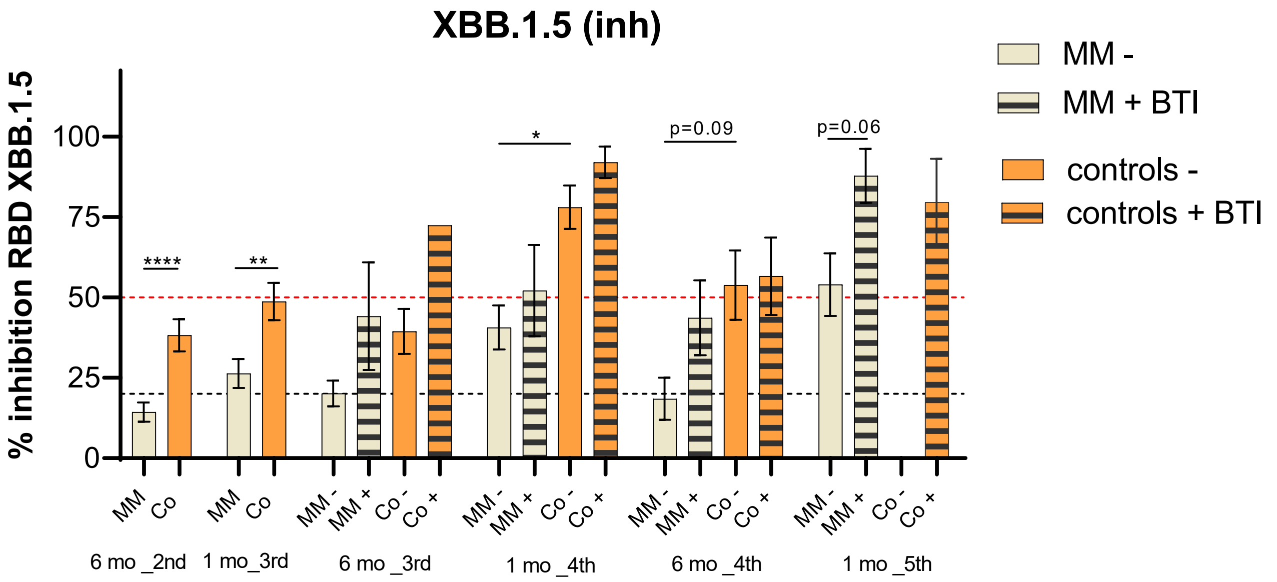
XBB.1.5 (inh)
% inhibition RBD XBB.1.5
0
25
50
75
100
****
**
*
p=0.09
p=0.06
MM
Co
MM
Co
MM -
MM +
Co -
Co +
MM -
MM +
Co -
Co +
MM -
MM +
Co -
Co +
MM -
MM +
Co -
Co +
6 mo _2nd
1 mo_3rd
6 mo _3rd
1 mo _4th
6 mo _4th
1 mo _5th
MM -
MM + BTI
controls -
controls + BTI

Supplementary Figure S3: *Leukocyte and lymphocyte counts pre-vaccination*

Leukocytes (white blood cells, WBC) and lymphocytes were measured in EDTA whole blood with SYSMEX XP-300 differential hematology analyzer in absolute numbers; A) WBC in peripheral blood (10^3/µl); B) lymphocytes as percentage of differential leukocyte count; C) absolute lymphocytes (10^3/µl) in the two investigated groups; lines represent median and interquartile range. Ab high responders were defined as >2500 BAUs/mL one month after second dose and >295 BAUs/mL after six months, and low responders as <1600 BAUs/mL one month and <295 BAUs/mL six months after second dose.
Abbreviations: MM, multiple myeloma; high, MM high-responders (MM patients with SCT +/- immunomodulatory therapy); low, MM low-responders (anti-CD38 treated MM patients); SCT, stem cell transplant.

Unpaired t-test or Mann Whitney test

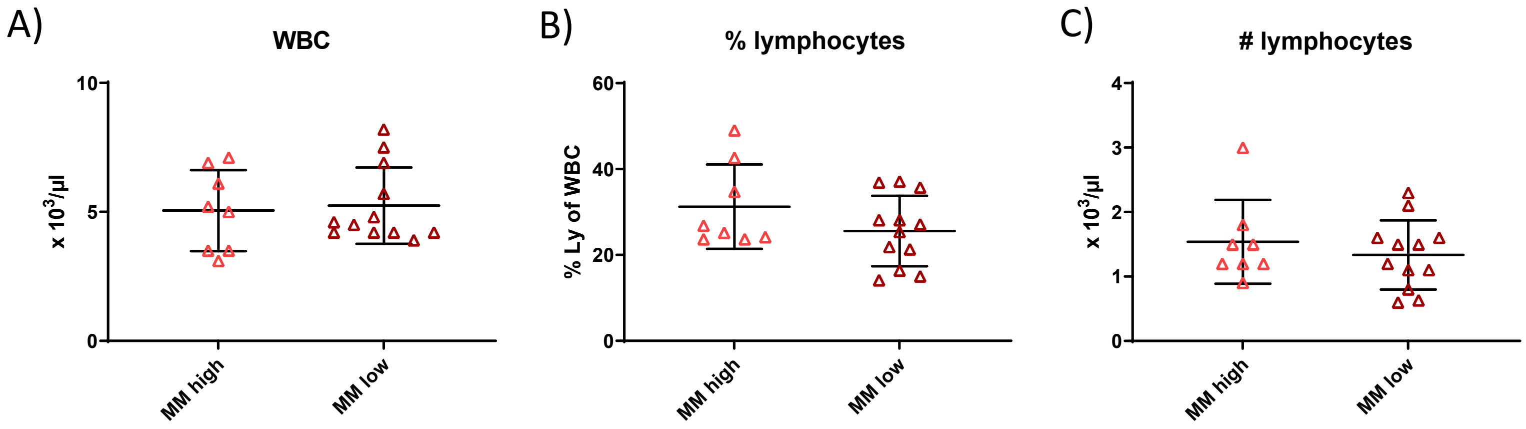

Supplementary Figure S4: *Quantification of T lymphocytes and the CD4, CD8 and NK-T-cell subset pre-vaccination*

PBMC obtained prior to vaccination were stained with fluorochrome-labeled mAbs and analyzed on a BD FACS Canto II flow cytometer. Quantification of CD3+ T cells A) as % of lymphocytes and B) as absolute numbers (10^2/µl); CD4 T cells (CD3+/CD4+) C) as % of total CD3+ T cells and D) in absolute numbers (10^2/µl); CD8 T cells (CD3+/CD8+) E) as % of total CD3+ T cells and F) in absolute numbers (10^2/µl); and CD3+/CD4-/CD8- NK-T cells G) calculated as % of lymphocytes and H) as absolute numbers (10^2/µl) in the two groups measured prior to vaccination; lines represent median and interquartile range; blue triangles in MM low group indicate patients receiving anti-CD38 mAb. Abbreviations: MM, multiple myeloma; high, MM high-responders (MM patients after SCT +/- immunomodulatory therapy); low, MM low-responders; SCT, stem cell transplant.

Unpaired t-test or Mann Whitney test; $**p \leq 0.01$; $*p \leq 0.05$

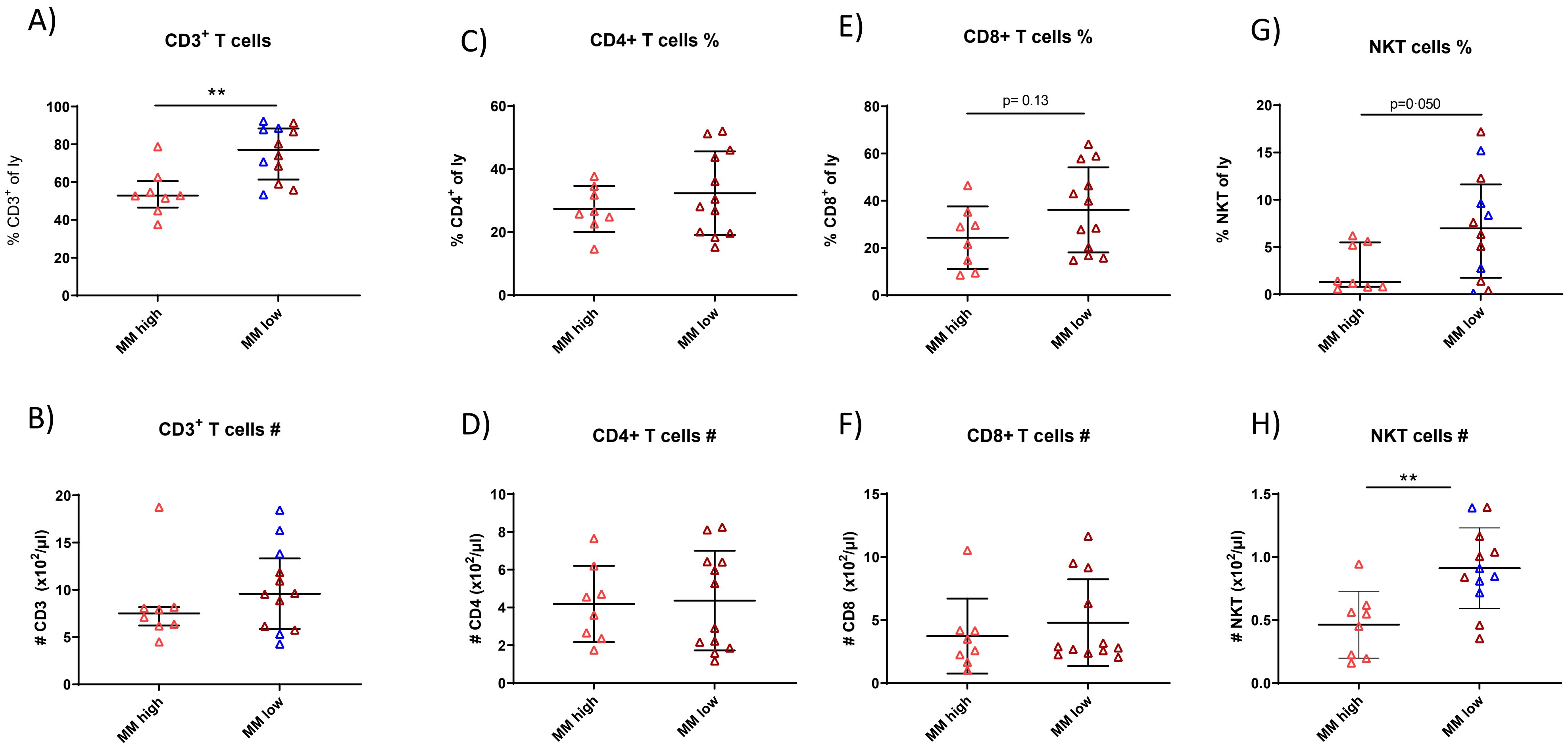

Supplementary Figure S5: *Quantification of B lymphocytes, immature transitional B cells and plasmablasts pre-vaccination*

Quantification of total CD19+ B-cells A) as % of lymphocytes and B) as absolute numbers (10^2/µl) calculated based on differential leukocyte counts in peripheral blood; quantification of immature transitional B cells (CD19+/CD24 ^{high}/CD38 ^{high}) C) as percentages of total CD19+ B cells and D) as absolute numbers (10^2/µl); quantification of plasmablasts (CD19+/CD27++/CD38 ^{high}) E) as percentages of total CD19+ B cells and F) as absolute numbers (10^2/µl) in the two investigated groups, measured prior to vaccination; lines represent median and interquartile range; blue triangles in MM low group indicate patients receiving anti-CD38 mAb.
Abbreviations: MM, multiple myeloma; high, MM high-responders (MM patients with SCT +/- immunomodulatory therapy); low, MM low-responders; SCT, stem cell transplant.

Unpaired t-test or Mann Whitney test; ***p ≤ 0.001; **p ≤ 0.01; *p ≤ 0.05

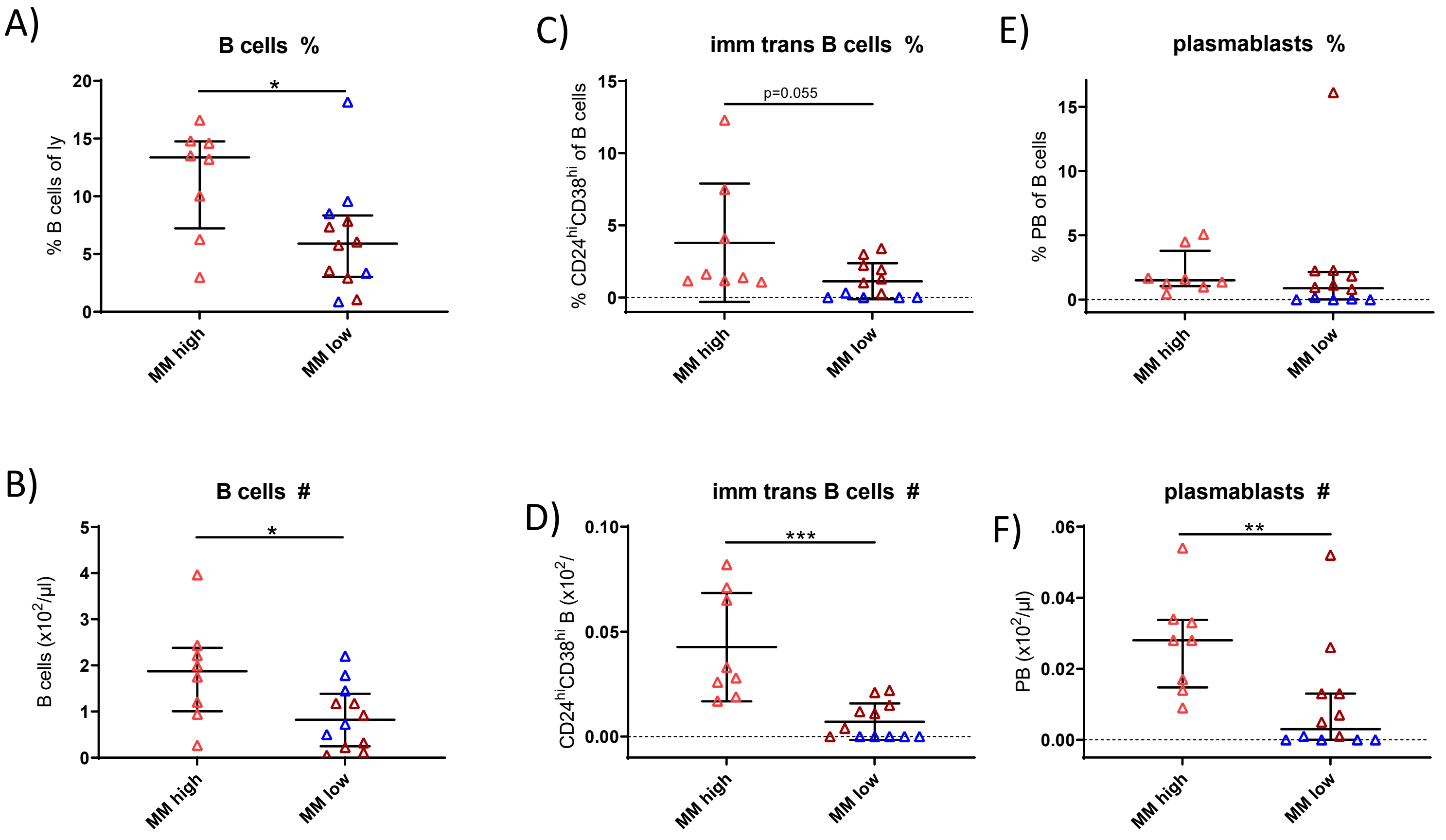

Supplementary Figure S6: *Quantification of NK cell subset pre-vaccination*

Quantification of NK cells A) calculated as % of lymphocytes; NK = (100 % ly – [% CD3+ T cells + % CD19+ B cells]) and B) as absolute numbers (10^2/µl) in the two investigated groups measured prior to vaccination; lines represent median and interquartile range; blue triangles in the MM low group indicate patients receiving anti-CD38 mAb. Abbreviations: MM, multiple myeloma; high, MM high-responders (MM patients with SCT +/- immunomodulatory therapy); low, MM low-responders (anti-CD38 treated MM patients); SCT, stem cell transplant.

Unpaired t-test or Mann Whitney test; **p ≤ 0.01

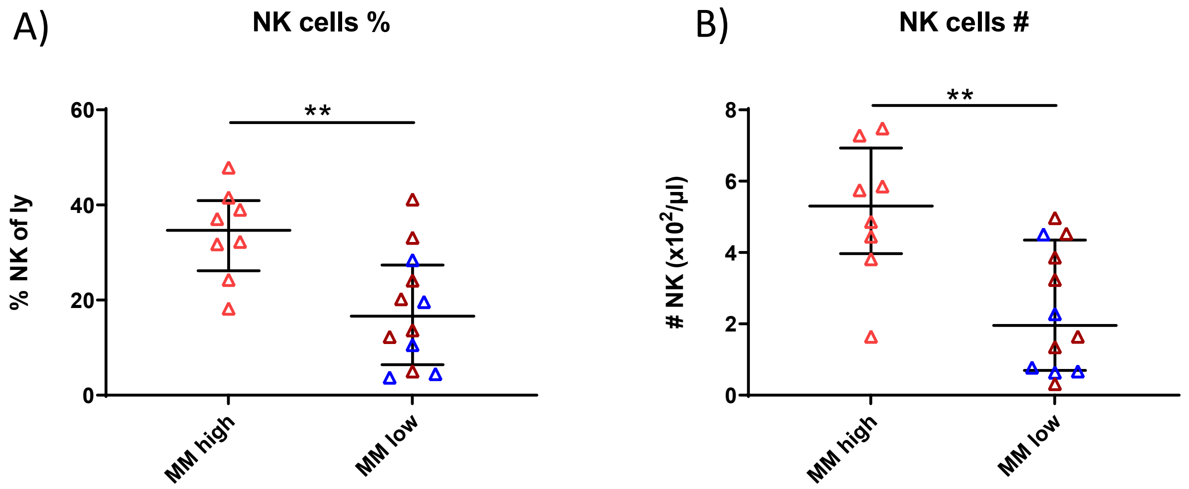

Supplementary Figure S7: *Kinetics of S1-specific IgG, S-specific memory B cells and plasmablasts in healthy controls*

Kinetic of S1-specific IgG (in BAU/mL), S-protein-specific memory B cells (as % of total B memory cells) and plasmablasts (PB; as percentages of total CD19+B cells) determined before vd1 and either one week (S-specific B memory and PB) or one month (S1-specific IgG) after vd2, one and six months after vd3, one and six months after vd4 in healthy controls. (n= 6), data points represent arithmetic mean with SEM.

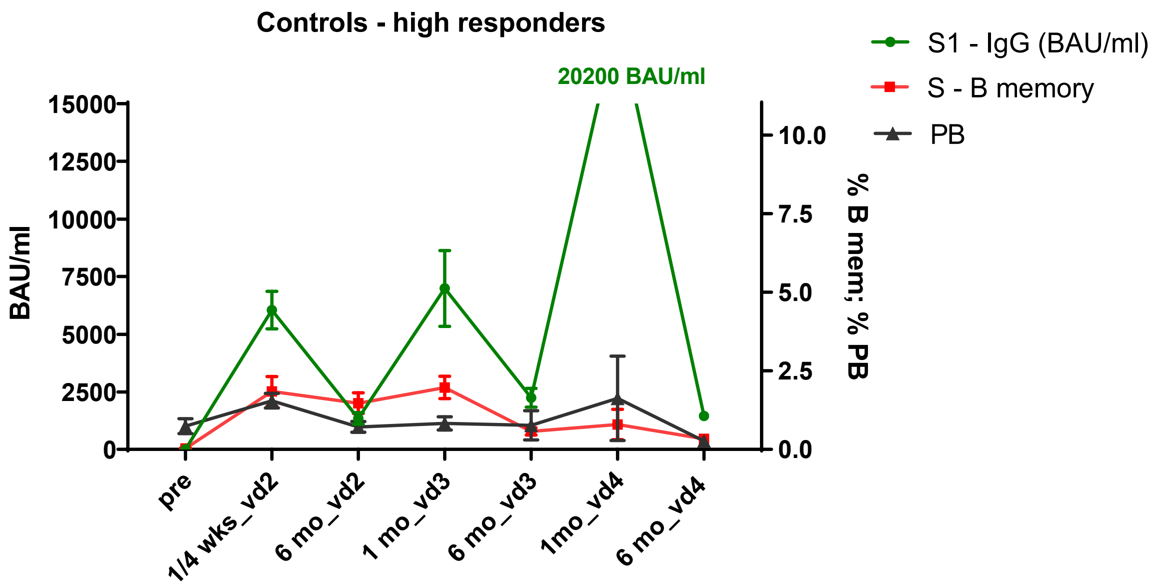

Abbreviations: BAU, binding antibody units; IgG, immunoglobulin G; PB, plasmablasts; S1, SARS-CoV-2 spike protein 1; SEM, standard error of the mean; vd, vaccine dose.

Supplementary Figure S8: *Correlations of-hu-1 S1 specific IgG Abs with concentrations of cytokines IFN-γ and IL-2 in PBMC culture supernatants*

Spearman rank correlations (r_s) of hu-1 S1-specific IgG (in BAU /mL) and cytokines (pg/ml) in MM patients for IL-2 A) six months after the vd2, B) one month after vd3, C) six months after the vd3, and D) one month after the vd4, and for IFN-γ E) six months after the vd2 dose F) one month after vd3 G) six months after vd3, and H) one month after vd4; in controls for IL2 I) six months after vd2, and J) one month after vd3 and for IFN-γ K) six months after vd2, and L) one month after vd3.

Abbreviations: Abs, antibodies; BAU, binding antibody units; CO, controls; MM, multiple myeloma; n.s.; not significant, vd, vaccine dose.

p values are indicated in graphs. ****$p \leq 0.0001$; *$p \leq 0.05$

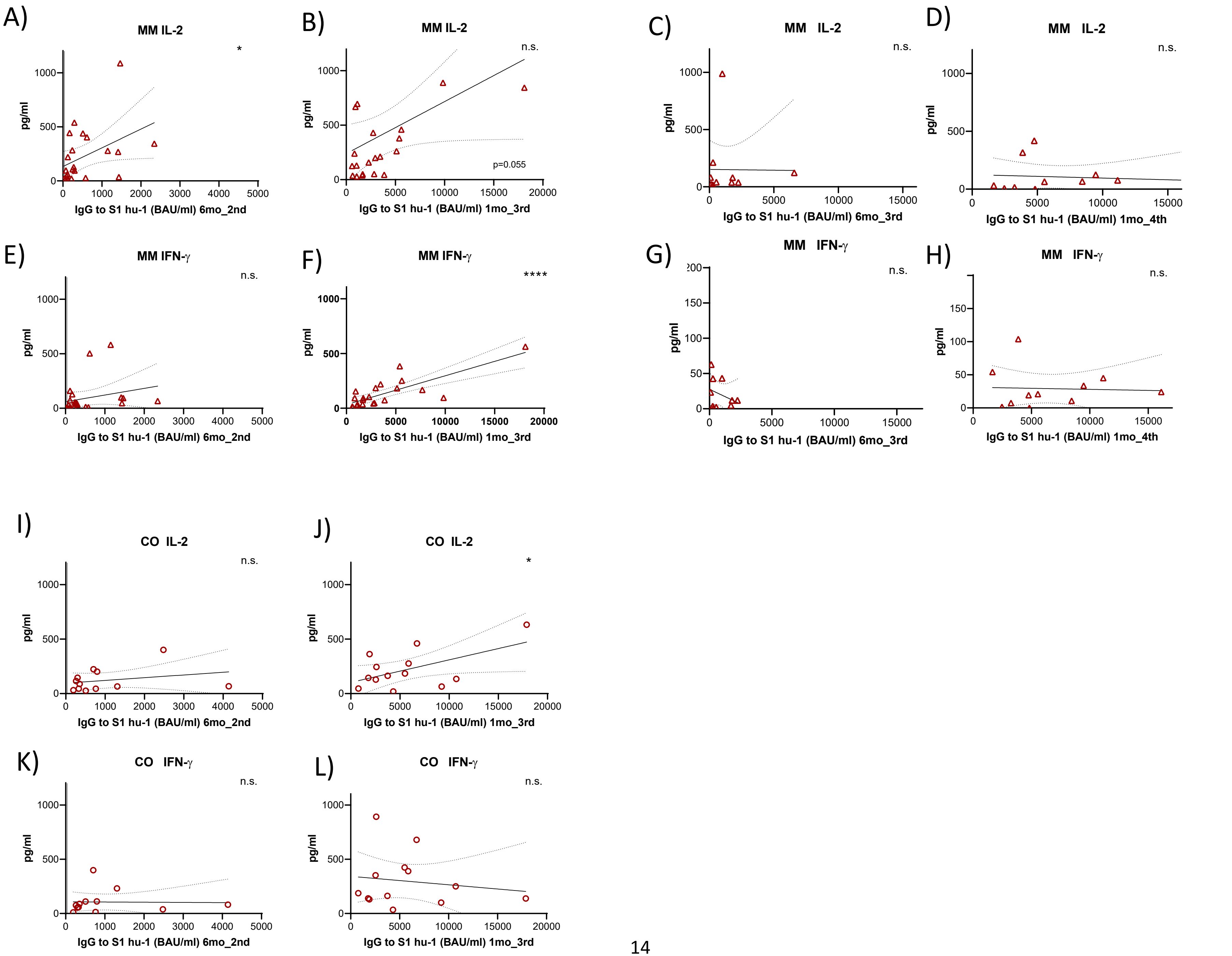
A)
MM IL-2
*
pg/ml
IgG to S1 hu-1 (BAU/ml) 6mo_2nd
B)
MM IL-2
n.s.
p=0.055
pg/ml
IgG to S1 hu-1 (BAU/ml) 1mo_3rd
C)
MM IL-2
n.s.
pg/ml
IgG to S1 hu-1 (BAU/ml) 6mo_3rd
D)
MM IL-2
n.s.
pg/ml
IgG to S1 hu-1 (BAU/ml) 1mo_4th
E)
MM IFN-γ
n.s.
pg/ml
IgG to S1 hu-1 (BAU/ml) 6mo_2nd
F)
MM IFN-γ
****
pg/ml
IgG to S1 hu-1 (BAU/ml) 1mo_3rd
G)
MM IFN-γ
n.s.
pg/ml
IgG to S1 hu-1 (BAU/ml) 6mo_3rd
H)
MM IFN-γ
n.s.
pg/ml
IgG to S1 hu-1 (BAU/ml) 1mo_4th
I)
CO IL-2
n.s.
pg/ml
IgG to S1 hu-1 (BAU/ml) 6mo_2nd
J)
CO IL-2
*
pg/ml
IgG to S1 hu-1 (BAU/ml) 1mo_3rd
K)
CO IFN-γ
n.s.
pg/ml
IgG to S1 hu-1 (BAU/ml) 6mo_2nd
L)
CO IFN-γ
n.s.
pg/ml
IgG to S1 hu-1 (BAU/ml) 1mo_3rd

Supplementary Data S1: *Inclusion and Exclusion criteria*

Inclusion criteria

- >=18 years
- Monoclonal gammopathy of unknown significance (MGUS) diagnosis
- Multiple myeloma (MM) diagnosis with or without immunosuppressive/immunomodulatory therapy
- No previous SARS-CoV-2 vaccination

Exclusion criteria

- Are not willing to get mRNA SARS-CoV-2 vaccination
- If female, are pregnant or lactating

If belonging to the healthy control group, are immunosuppressed (suffer from or have a history of immune mediated diseases, long-term use of corticosteroids, haemodialysis, chronic renal insufficiency, liver cirrhosis Child-Pugh class C, haematooncologic malignant disease, solid organ transplant).

Supplementary Data S2: *Fluorochrome-conjugated monoclonal Abs for flow-cytometric analysis of B and T cell panel:*

The following mAbs were used: anti–human CD3 PerCP-Cy5.5 (clone Ucht1), anti–human CD4 APC-H7 (clone L200), anti–human CD8 APC (clone RPA-T8), anti–human CD45RA BV421 (clone HI100), anti–human CD19 FITC (clone HIB19), anti–human CD27 PE (clone L128), anti–humanCD38 PerCP-Cy5.5 (clone HIT2), anti–human CD24 BV421(clone ML5), anti–human CD10 BV510 (clone HI10a), anti–human immunoglobulin D (IgD) PE-Cy7 (clone IA6-2), all from BD Biosciences; anti–human chemokine receptor 7 (CCR7) FITC (clone 150503) was obtained from R&D Systems, Inc. (Minneapolis, MN, USA). Dead cells were excluded by using fixable viability dye eFluor-780 (B panel) and eFluor-506 (T panel, both from eBioscience, now Thermo Fisher Scientific). Natural killer T cells were characterized as CD3+/CD4−/CD8− cells and were calculated as the difference of [CD3+/CD4+ plus CD3+/CD8+ T cells] to total CD3+ T cells as % of lymphocytes. NK cells were calculated as percentages of lymphocytes according to (% NK = 100 % lymphocytes – [% CD3+ T cells + % CD19+ B cells]).
